# Liposomal antimicrobials in the fight against bacterial and fungal pathogens: Clinical successes and development challenges

**DOI:** 10.1016/j.ijpx.2025.100478

**Published:** 2025-12-23

**Authors:** Hussein T. Kenaan, Ross M. Duncan, Wafa T. Al-Jamal, David S. Jones, Gavin P. Andrews, Brendan Gilmore, Vanessa Yardley, Nicola Farrington, Katharine E. Stott, David Lawrence, Joseph N. Jarvis, Thomas S. Harrison, Stephen Robinson, Isabela Ribeiro, William Hope, Yiwei Tian

**Affiliations:** a School of Pharmacy, Queen's University Belfast, 97 Lisburn Road, Belfast, UK; b Faculty of Infectious and Tropical Diseases, London School of Hygiene and Tropical Medicine, London, UK; c Department of Antimicrobial Pharmacodynamics and Therapeutics, University of Liverpool, Liverpool, UK; d Liverpool University Hospitals NHS Foundation Trust, Liverpool, UK; e Department of Clinical Research, Faculty of Infectious and Tropical Diseases, London School of Tropical Medicine, London, UK; f Botswana Harvard Health Partnership, Gaborone, Botswana; g School of Pathology, Faculty of Health Sciences, University of the Witwatersrand, Johannesburg, South Africa; h Institute of Infection and Immunity, City St George's University London, London, UK; i MRC Centre for Medical Mycology, University of Exeter, Exeter, UK; j Drugs for Neglected Diseases initiative, Geneva, Switzerland

**Keywords:** Antimicrobial resistance, Invasive fungal infections, Liposomal amphotericin B, Liposomal amikacin, Biological barriers, Nanomedicine manufacturing challenges

## Abstract

Bacterial, fungal, and protozoan infections pose a rapidly escalating threat to global health, exacerbated by the rise in antimicrobial resistance. Current therapies against microbial pathogens are limited by high systemic toxicity and poor drug solubility. Liposomal formulations (spherical vesicles composed of lipid bilayers) have demonstrated remarkable clinical potential in addressing these concerns, as evidenced by the marketed products AmBisome® and Arikayce®. These products, which deliver amphotericin B via parenteral injection and amikacin via inhalation, exemplify how liposomes effectively mitigate drug-associated toxicity, enhance therapeutic efficacy, and overcome the biological barriers inherent to infection sites, including complex microbial biofilms, mucosal interfaces, or the blood–brain barrier. Complementary insights from anticancer research indicate that strategic manipulation of liposomal composition and structure can enhance their therapeutic potential. Adjustments in lipid charge, fluidity, and PEGylation, in particular, highlight their versatility and broad applicability for antimicrobial drug delivery. Liposomal antimicrobials can modulate pharmacokinetic profiles, achieve targeted release at sites of infection, and increase local drug concentrations, which are key advantages over conventional treatments. Despite these therapeutic advances, successful clinical translation and widespread adoption of liposomal antimicrobials remain highly dependent on overcoming existing technological and manufacturing challenges. This review emphasises the need for a paradigm shift within liposomal antimicrobial development, encouraging progression from initial research and development toward scalable, reproducible, and economically viable commercial manufacturing platforms. This transition is essential not only for ensuring the global accessibility and affordability of existing therapies but also for expanding the development of clinically relevant liposomal antimicrobial nanomedicines.

## Highlights

• Liposomal antimicrobials can reduce the toxicity and enhance efficacy against bacterial and fungal infections through barrier penetration and extended release.
• We advocate for simple liposomal formulation design and scalable manufacturing technologies to ensure affordable global access and to accelerate progress in curbing AMR.

## Graphical abstract

**Figure:**
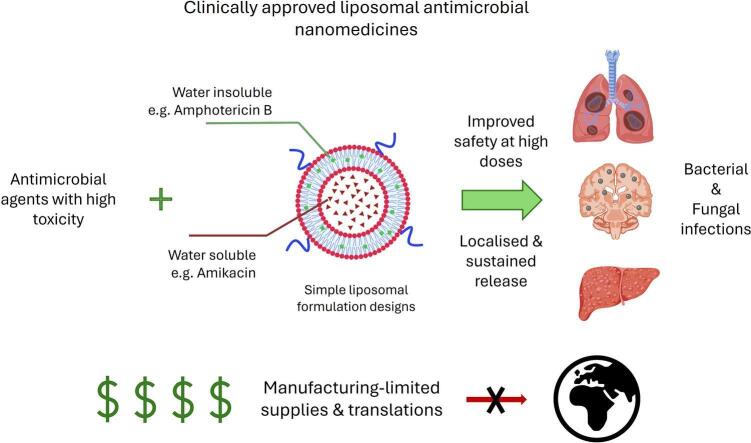

## 1 Introduction

Bacterial, fungal and protozoal diseases pose a threat to global health, with an estimated 7.7 million deaths linked to 33 bacterial species across 11 infectious syndromes in 2019 alone, most frequently involving *Staphylococcus aureus* and *Escherichia coli* (Baig et al., 2024; Ikuta et al., 2022). Furthermore, approximately 2.5 million deaths are attributable to fungi annually, with invasive and chronic pulmonary aspergillosis having the most significant mortality, followed by bloodstream and invasive candidiasis (Denning, 2024). The global morbidity figure may increase to 11 million by 2050, given bacterial and fungal resistance and comorbidities, driven by the widespread dissemination of resistant strains, including those of *E. coli* and Candida spp. (Lockhart et al., 2017; Naghavi et al., 2024). Healthcare systems are suffering from the immense dual threat of large-scale, widespread bacterial infections and the high mortality rate of fungal diseases in immunocompromised patients, highlighting the necessity for coordinated action. To address this, the UN, WHO, leading charities, and government agencies are now working together to form critical strategic partnerships to curb preventable deaths and antimicrobial resistance. In an attempt to encourage research into the previously overlooked need for fungal antimicrobial resistance (AMR) control, the WHO released the first-ever fungal priority pathogen list (WHO, 2022b). Following this, the UN declared its target to achieve a 10 % reduction in annual deaths caused by drug-resistant infections by 2030 and highlighted the need for a revision of the global action plan for AMR by 2026 (WHO, 2024). The Global Antibiotic Research and Development Partnership (GARDP), set up by the Drugs for Neglected Diseases Initiative (DNDi), alongside the WHO, recently described the lack of access to appropriate treatments for carbapenem-resistant gram-negative bacteria in low- and middle-income countries (LMICs) (Mishra et al., 2025), further highlighting the challenges associated with managing AMR on a global scale.

To address bacterial AMR, the key focus has been on ESKAPE pathogens (*Enterococcus faecium*, *Staphylococcus aureus*, *Klebsiella pneumoniae*, *Acinetobacter baumannii*, *Pseudomonas aeruginosa* and *Enterobacter* spp.), which are considered the most virulent and drug-resistant bacteria from a clinical perspective and pose a significant challenge to current antibiotic regimens, resulting in frequent reports of treatment failure, infection relapse, and increased mortality rates (Miller and Arias, 2024). Of critical concern are *P. aeruginosa* and *A. baumannii*, two Gram-negative organisms for which there are increasing reports of multidrug-resistant (MDR) and extensively drug-resistant (XDR) strains (del Barrio-Tofiño et al., 2019; Ibrahim et al., 2021; Mirzaei et al., 2020). Both are associated with nosocomial infections, including pneumonia, bacteraemia, urinary tract infections (UTIs), and burn-wound infections (Driscoll et al., 2007; Eliopoulos et al., 2008), accompanied by mortality rates of 46 % for hospital-acquired *P. aeruginosa* infections (Raman et al., 2018) and 41 % for *A. baumannii* infections within intensive care units (Corbella et al., 1996). Vancomycin-resistant enterococci (VRE) and methicillin-resistant *Staphylococcus aureus (MRSA)*, two gram-positive ESKAPE pathogens*,* are also of high concern. The latter was responsible for the highest absolute number of deaths caused by any pathogen–drug combination analysed (Murray et al., 2022). Although VRE has a lower incidence than other ESKAPE pathogens (Willems et al., 2023), its mortality rate remains high. It is comparable to that of pathogens with higher incidence rates (De Oliveira et al., 2020). For fungal AMR, two Candida species, *C. albicans* and *C. auris*, have been identified as critical priorities, with others in the genus categorised as high priority by the WHO (WHO, 2022b). Mortality rates of 40 % have been reported for bloodstream *C. albicans* infections, despite attempted antifungal intervention (Lee et al., 2020). Multidrug resistance is a significant concern in *C. auris*, with 41 % of strains resistant to two major antifungal classes and 4 % resistant to all three major classes (Lockhart et al., 2017), resulting in mortality rates as high as 60 % (Chowdhary et al., 2017). Furthermore, an increase in azole resistance in *Aspergillus fumigatus* has been reported, suspected to be due to the agricultural use of fungicides (Meis et al., 2016). In cases where invasive aspergillosis does not respond to initial azole treatments, reported mortality rates can range from 50 to 100 % (Verweij et al., 2016). Through rapid genetic mutation and the exchange of mobile genetic elements, the spread of AMR genes is occurring at an alarming rate (De Oliveira et al., 2020). Resistance to virtually all major classes of antibacterial and antifungal agents has been documented, and many MDR organisms have been identified (Basak et al., 2016; Lockhart et al., 2017). Furthermore, the biofilm-forming ability of microorganisms is increasingly recognised as a significant determinant of antimicrobial tolerance and infection persistence (Bowler et al., 2020; Ramage et al., 2025). An extensive discussion of the various drug resistance/tolerance mechanisms utilised by bacteria and fungi can be found in the reviews by Darby et al. (2023) and Perlin et al. (2017), respectively.

Furthermore, the discovery of natural antimicrobials has decreased exponentially over the past half-century, partly owing to the requirement for larger strain libraries, which surpass what is considered practical within the industry (Baltz, 2007). Even synthetic antimicrobials, such as fluoroquinolones, have lost the race with bacteria due to resistance mechanisms being passed between species (Lewis, 2017). The lack of new, effective antimicrobial agents, along with the increasing incidence of resistance to existing drugs, has driven AMR research toward innovative drug-delivery systems, including antimicrobial conjugates with siderophores, peptides, and antibodies, as well as nanocarriers such as liposomes and polymeric nanoparticles.

Limited solubility has long been a significant limitation of antimicrobials, such as tetracyclines and several macrolides, as well as virtually all azoles and polyenes, which can render them incompatible with simple intravenous injection or result in suboptimal exposure in target tissue. Tetracycline hydrochloride salt is used to substantially increase solubility in water, rapidly increasing absorption after oral administration without increasing toxicity (Varanda et al., 2006). Similarly, amphotericin B (AmB) is complexed with deoxycholate in its conventional formulation and voriconazole is complexed with sulfobutyl ether-beta-cyclodextrin (SBECD) to increase its solubility; however, this complex is accompanied by significant systemic toxicity issues, including nephrotoxicity (Bellmann and Smuszkiewicz, 2017). In addition to solubility, rapid hepatic metabolism can independently contribute to toxicity. For example, the rapid metabolism of voriconazole leads to significant drug–drug interactions involving cytochrome P450 enzymes, further increasing its potential toxicity (Theuretzbacher et al., 2006). Such solubility-independent toxicity remains a critical concern for several antimicrobial agents, including aminoglycosides, glycopeptides, and oxazolidinones, particularly in clinical settings where these agents are used for extended periods if initial treatment fails.

To overcome the issues described with drug delivery, drugs can be encapsulated within spherical liposomal particles, which have revolutionised the use of many chemotherapy drugs, as well as the antimicrobials AmB and amikacin (AMK). Some of the most important benefits of using liposomes to deliver drugs are increased solubility, extended plasma half-life, and increased delivery to target tissues, all of which contribute to improved safety of the treatment (Gill et al., 1996; Glantz et al., 1999; Griffith et al., 2018; Meunier et al., 1991; Stewart et al., 1998). Other potential benefits include increasing the intracellular uptake of drugs in gram-negative bacteria via liposome–membrane fusion (Alipour et al., 2008), protecting cargo from drug-modifying enzymes such as β-lactamases (Di Rocco et al., 1992; Nacucchio et al., 1988), and slowing drug efflux rates (Khameneh et al., 2015). In combating AMR, liposomes provide a versatile strategy for enhancing drug efficacy (Rukavina and Vanić, 2016) and overcoming tolerance mechanisms associated with biofilm formation and intracellular survival in host cells (Zhang et al., 2018). Their ability to engage with biofilm components (Makhlouf et al., 2023) and their potential to administer synergistic drug combinations (Schiffelers et al., 2001; Wallace et al., 2013) hold promise for addressing both bacterial and fungal diseases. While other nanocarrier systems have been explored for antimicrobial delivery, their clinical and regulatory progress remains limited. Lipid nanoparticles have shown promise in certain therapeutic areas but have had limited success in antibacterial or antifungal applications (Kumar, 2025), owing to their limited interaction with microbial membranes relative to liposomes' fusogenic potential (Zhang et al., 2020). Similarly, nanoemulsions have been investigated as carriers for antimicrobials; however, no antibacterial or antifungal nanoemulsion formulation has achieved regulatory approval or commercial success, making their clinical translation difficult to realise (Garcia et al., 2022). Micellar systems have also demonstrated higher toxicity and inferior safety profiles, as evidenced by early AmB micellar formulations being superseded by the safer liposomal AmBisome® (Hamill, 2013). Apart from Arikayce® and AmBisome®, no other lipid nanoparticle or nanoemulsion formulations have reached the antimicrobial market (Thorn et al., 2021). In contrast, liposomes remain the most versatile and clinically validated nanocarriers, capable of encapsulating both hydrophilic and hydrophobic drugs and supported by established regulatory frameworks and successful marketed products (Đorđević et al., 2022; Gomez and Hosseinidoust, 2020; Thorn et al., 2021; Zhang et al., 2020).

Liposomal antimicrobials can overcome common toxicity and tissue distribution challenges associated with conventional formulations, with two standout examples, AmB and AMK, at the forefront of this development. In this review, we provide an in-depth examination of these key products, including their innovative formulations and associated clinical benefits. By using AmBisome® and Arikayce® as clinical case examples, we aim to connect mechanistic insights, pharmacokinetics, and safety with practical aspects of composition, manufacturing, and sterilisation, and to consider how lessons from these products and from anticancer liposomal nanomedicines can inform the rational design of future liposomal antimicrobial candidates. This integrated and translational perspective is intended to complement existing reviews on AMR and nanomedicine by focusing on what can be learned from licensed liposomal antimicrobials and how this knowledge can guide more scalable and globally relevant development.

## 2 Antifungal drug amphotericin B

**Fig. 1:**
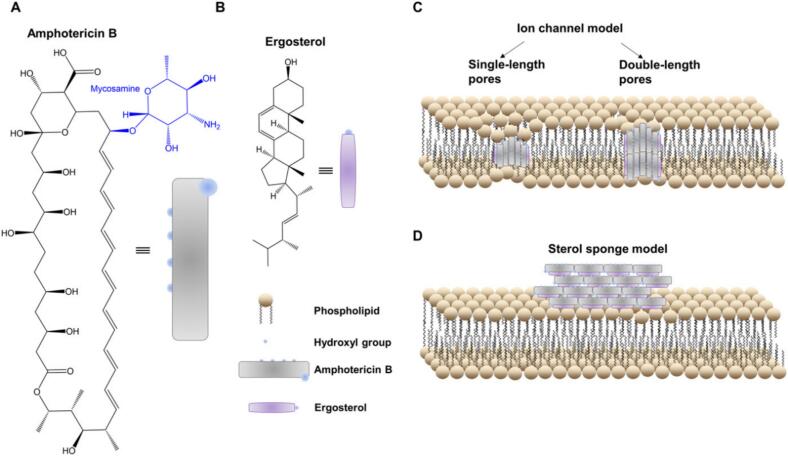
Proposed AmB mechanisms of action on fungal cells. Chemical structures of AmB (A) and ergosterol (B). (C) Ion channel model. (D) Sterol sponge model; adapted from Dong et al. (2021).

Since its commercialisation in the 1950s, AmB has played a prominent role in treating systemic fungal infections and protozoan diseases, such as leishmaniasis, and remains the agent with the broadest antifungal spectrum; reports of antimicrobial resistance are rare (Anderson et al., 2014; Fernández-García et al., 2017). AmB is a polyene antibiotic that was first isolated from bacterial cultures of *Streptomyces nodosus* (Torrado et al., 2008). It is a type of polyene macrolide (Cavassin et al., 2021) and is characterised by its amphiphilic and amphoteric properties (Fig. 1A). These characteristics stem from the hydrophobic and hydrophilic regions of its lactone ring, coupled with ionisable carboxyl and amine groups (Torrado et al., 2008). According to the biopharmaceutical classification system (BCS), AmB is classified into class IV owing to its poor aqueous solubility at physiological pH and poor permeability (Wang et al., 2021). The oral bioavailability of AmB has been reported to be extremely low (0.2–0.9 %) (Serrano and Lalatsa, 2017).

AmB acts by binding to fungal cell ergosterols and promoting leakage of cell contents. Ergosterol is also present in certain parasites, such as *Leishmania*. While the complex mechanisms of action of AmB are still unclear, it has also been suggested that the drug can intercalate into the plasma membrane, forming pores that alter the permeability of Na^+^, K^+^, and H^+^, leading to the loss of carbohydrates and proteins and ultimately cell death (Fig. 1C) (Mesa-Arango et al., 2012; Palacios et al., 2011). More recent research also suggests that AmB can attack fungi by forming extramembranous, sponge-like aggregates that extract ergosterol from lipid bilayers rather than through its other role in creating membrane-inserted ion channels (Fig. 1D) (Anderson et al., 2014; Lewandowska et al., 2021; Revie and Cowen, 2021).

These two proposed mechanisms differ in their descriptions of the interaction of AmB with fungal phospholipids: in the ion channel model, AmB molecules assemble parallel to the phospholipid acyl chains, whereas in the sterol sponge model, they align orthogonally (Anderson et al., 2014; Dong et al., 2021). Recent studies indicate that the pore formation and sterol extraction models are not mutually exclusive; instead, both mechanisms likely contribute simultaneously to the antifungal activity of AmB (Delhom et al., 2020). Notably, the prevailing mechanism appears to be dependent on the local AmB concentration at the fungal cell membrane; lower concentrations favour pore formation, whereas higher concentrations promote sterol extraction or a combination of both (Wang et al., 2024).

AmB can act as a fungicidal or fungistatic agent depending on the dose and fungal strain (Campoy and Adrio, 2017; Fernández-García et al., 2017). In addition to its affinity for fungal ergosterol, AmB may bind less selectively to mammalian cell cholesterol. This characteristic is responsible for its severe kidney, heart, and blood cell toxicity in clinical practice (Cavassin et al., 2021; Grela et al., 2018). AmB toxicity manifests as acute infusion-related reactions, anaemia, thrombocytopenia, and dose-dependent nephrotoxicity, which typically limits the maximum tolerated dose (Hamill, 2013; Shigemi et al., 2011). Owing to its broad-spectrum fungicidal activity against yeasts and filamentous fungi, AmB is considered a first-line treatment for invasive fungal infections, including cryptococcal meningitis, pulmonary aspergillosis, and invasive mucormycosis (Cavassin et al., 2021; Chang et al., 2017; Cornely et al., 2019; Hoenigl et al., 2018). AmB can also be used preemptively in situations with a high risk of fungal infection, particularly among patients with neutropenia or those undergoing chemotherapy or stem cell and organ transplantation (Chen et al., 2023). In addition to its antifungal activity, AmB has been well established for the treatment of visceral leishmaniasis (Meyerhoff, 1999). All currently available commercial formulations of AmB are designed for intravenous administration and include micellar dispersions, lipid complexes, liposomes, and colloidal dispersions, each with distinct efficacy and safety profiles.

### 2.1 AmB deoxycholate (Fungizone®)

The easiest way to address AmB's poor water solubility was to formulate it as a colloidal suspension, which was first commercialised in 1958 as Fungizone® with sodium deoxycholate (a bile salt surfactant) at a molar ratio of 1:2 (Faustino and Pinheiro, 2020; Serrano et al., 2013). The Fungizone® dispersion contains micelles of approximately 40 nm (Dupont, 2002; Fernández-García et al., 2017; Serrano et al., 2013). Clinically, intravenous infusion of Fungizone® should be administered over 2–6 h, depending on the dose, to reduce AmB-related infusion side effects (Fleury et al., 2016). Fungizone® can cause an acute reaction, including high fever, chills, hypotension, nausea, vomiting, arrhythmias, and rash, 1–3 h following infusion (Laniado-Laborín and Cabrales-Vargas, 2009; Min et al., 2015). Since the optimal dosage has not been established, it should be personalised and adjusted based on the patient's clinical condition. The dose-related toxicity of AmB in Fungizone® typically limits the maximum tolerated dose to 0.7–1.0 mg/kg per day, a dosage that is suboptimal for achieving clinical success against many invasive fungal pathogens in normal and immunocompromised patients (Hamill, 2013). The efficacy of Fungizone® has been heavily affected by its severe and potentially fatal toxicity (Janknegt et al., 1992).

Despite this, Fungizone® remains widely used in clinical practice, particularly in LMICs, owing to its **relatively low cost** and critical role in treating life-threatening fungal infections (Falci et al., 2011; Lee et al., 2024). Moreover, it remains comparable to lipid-based formulations in terms of microbiological efficacy (Kleinberg, 2006; Walsh et al., 1999). Despite the enhanced tolerability of lipid-based formulations, high prices (Olliaro et al., 2009) and complex manufacturing processes (Adler-Moore and Proffitt, 1993) limit their global use, particularly in low- and middle-income countries (Zia et al., 2017). Reports have suggested that using AmB deoxycholate in a continuous 24-h infusion can reduce nephrotoxicity while maintaining efficacy in patients with malignancies, stem cell transplant recipients, and HIV patients with cryptococcosis, thereby improving tolerability (Bellmann and Smuszkiewicz, 2017; Falci et al., 2011). To overcome the toxicity and dose limitations associated with Fungizone®, several lipid-based AmB formulations have been developed and approved by regulatory agencies such as the FDA and the EMA **since** the 1990s (Hamill, 2013; Serrano et al., 2013). Among the lipid-based formulations of AmB, Abelcet® and AmBisome® are currently available for clinical use, while Amphotec®/Amphocil® was previously marketed but has since been discontinued (Hamill, 2013; Min et al., 2015). Of the available lipid-based AmB products, AmBisome® has demonstrated the most favourable balance of efficacy, safety and pharmacokinetics, making it the focus of the next section.

### 2.2 Liposomal AmB

Liposomal AmB (LAmB), best represented by AmBisome®, is widely recognised for its superior safety profile, enabling high-dose delivery of AmB with reduced nephrotoxicity and improved pharmacokinetic properties. LAmB-targeted delivery can be achieved through prolonged circulation with reduced systemic clearance, passive accumulation at inflamed sites, and local phagocyte interactions that support retention and tissue redistribution (Adler-Moore and Proffitt, 2008; Liu et al., 2020c; Stone et al., 2016). In high-income countries, LAmB has largely replaced conventional AmB and other lipid-based formulations, becoming established as the preferred treatment for invasive fungal diseases in immunocompromised patients, despite its relatively high cost (Lee et al., 2024).

**Fig. 2:**
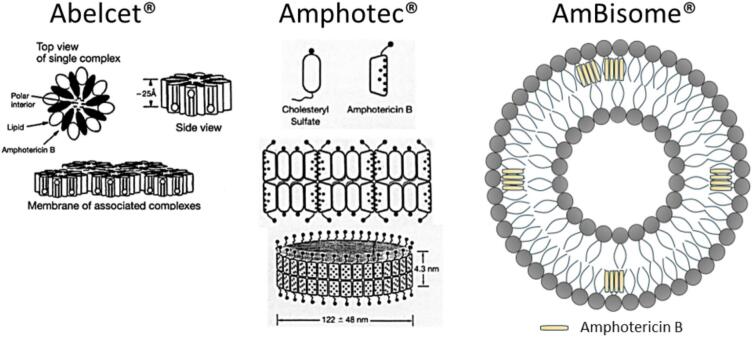
Structures of lipid-based AmB formulations; adapted from Min et al. (2015) with modifications.

As illustrated in Fig. 2, liposomes are spherical vesicles with a shell of one or more phospholipid bilayers that encapsulate an internal hydrophilic core and are distinct from other lipid-based AmB formulations (Bozzuto and Molinari, 2015a; Nisini et al., 2018b). Liposomes can serve as flexible carriers for different types of drugs and proteins: hydrophilic substances may be encapsulated in the aqueous core, whereas lipophilic substances such as AmB can be incorporated into the lipid bilayers (Slain, 1999; Wong-Beringer et al., 1998). Liposomes, as drug delivery vehicles, have a well-established track record of reducing the toxic side effects associated with anticancer medications such as doxorubicin and cytarabine (Garcia-Marco et al., 2009; Harris et al., 2002). In theory, encapsulating AmB within liposomes could increase its therapeutic index by facilitating its selective delivery to fungal cells while minimising its uptake by human cells (Wong-Beringer et al., 1998). The first LAmB, AmBisome®, was approved in the European market in 1989, but it was not until 1997 that it received FDA approval in the USA (Hamill, 2013).

AmBisome® is a unique liposomal formulation in which AmB is integrated into the bilayer part of unilamellar liposomal structures, with a mean particle size of less than 100 nm and a negative Zeta potential of approximately −40 mV (Liu et al., 2020b). It consists of hydrogenated soy phosphatidylcholine (HSPC), cholesterol, distearoylphosphatidylglycerol (DSPG), and AmB (2:1:0.8:0.4 mol ratio) (Schmidt et al., 1998). These lipids (DSPG, HSPC, and cholesterol) collectively have a high transition temperature and were selected to produce a formulation that would remain stable at 37 °C. Furthermore, DSPG was selected to enhance the stability of the AmB-containing bilayers. During liposome preparation, the hydration step is carried out at mildly acidic pH values (∼5.5), where the negatively charged headgroup of DSPG can form ionic interactions with the protonated amino group of AmB, enhancing drug retention within the bilayer (Rivnay et al., 2019; Stone et al., 2016; Svirkin et al., 2022). Cholesterol is added to increase the rigidity of the bilayer and retain AmB, as it has a binding affinity for AmB (Torrado et al., 2008). Every set of eight AmB molecules interacts with DSPG and cholesterol within the liposome, similar to the interaction with fungal ergosterol (Hillery, 1997); thus, AmB is tightly anchored in the liposomal bilayer because of these favourable interactions of the drug with the surrounding lipids (Schmidt et al., 1998). Other excipients, such as alpha-tocopherol (an antioxidant), are also included in the formulation, along with sucrose and sodium succinate as isotonic agents (Torrado et al., 2008). The formulation is supplied as a freeze-dried powder for reconstitution in water immediately before infusion (Torrado et al., 2008). The reconstituted formulation contains approximately 29 mg/mL of total lipid and 4 mg/mL AmB (Liu et al., 2020b).

The physicochemical properties of liposomal AmB preparations, including particle size, surface electrostatic charge, lipid bilayer rigidity, and the quantity of lipid material incorporated, crucially influence their pharmacokinetics and particle distribution (Janknegt et al., 1992). Notably, smaller vesicles, such as AmBisome®, often have an extended systemic circulation time because they are not readily recognised or taken up by components of the mononuclear phagocyte system. This decreased systemic clearance prolongs circulation, allowing vesicles to reach inflamed tissues where passive extravasation and local phagocyte interactions support retention and drug release. Within the liver, smaller vesicles can extravasate through fenestrations of the hepatic sinusoids, enabling interaction with hepatocytes and avoiding uptake by Kupffer cells. The significantly higher peak plasma concentration (C_max_), increased area under the concentration–time curve (AUC), and reduced volume of distribution (V_d_) observed with LAmB (Table 1) support the notion that this formulation may have the ability to achieve greater secondary tissue distribution, compared to other marketed AmB formulations, providing extended drug release at infection sites than other lipid-based formulations (Heinemann et al., 1997; Walsh et al., 1998). Increasing liposome bilayer rigidity can reduce permeability across the lipid membrane and prevent premature drug release. Cholesterol, for example, has been incorporated into some liposomal formulations, rendering them more resistant to degradation by preventing the exchange of liposomal components with plasma lipoproteins. The physicochemical properties of LAmB mentioned above have contributed to its favourable therapeutic index (Adler-Moore and Proffitt, 2002; Adler-Moore et al., 2016; Janknegt et al., 1992).

**Table 1:** Physical and pharmacokinetic properties of AmB formulations; adapted from Hamill (2013).

Property	AmB formulations			
	AmB deoxycholate (Fungizone®)	LAmB (AmBisome®)	ABLC (Abelcet®)	ABCD (Amphotec®)
Composition (molar ratio)	–	HSPC:cholesterol:DSPG 10:5:4	DMPC:DMPG 7:3	Cholesteryl sulfate
Structure	Micelles	Unilamellar spherical liposomes	Ribbons	Discs
AmB:lipid molar ratio	NA	1:9	1:3	1:1
Size (nm)	35	< 100	1600 - 11,000	110–140
Safe dose (mg/kg)	≤1	>10	≤5	≤5
C_max_ (μg/mL)	1.5–2.9	83 ± 35.2	1.7	2.9
AUC (μg·h/mL)	17.1–36	555 ± 311	14.0 ± 7.0	36
V_d_ (L/kg)	5.0 ± 2.8	0.16	131 ± 57.7	4.1
Cl (mL/h/kg)	38.0 ± 15.0	11.0 ± 6.0	436 ± 188	112

AUC: area under the concentration–time curve, Cl: clearance, C_max_: peak plasma concentration, DMPC: dimyristoyl phosphatidylcholine, DMPG: dimyristoyl phosphatidylglycerol, DSPG: distearoyl phosphatidylglycerol, HSPC: hydrogenated soy phosphatidylcholine, NA: not applicable, V_d:_ volume of distribution.

**Fig. 3:**
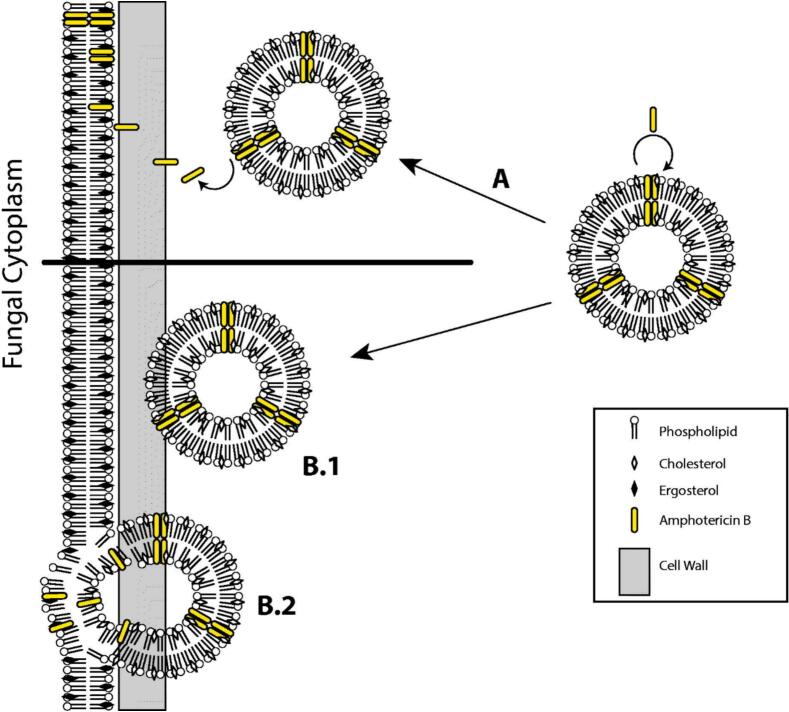
Proposed modes of interaction between LAmB and fungal cells: (A) classical model in which AmB dissociates from the liposomal bilayer upon perturbation at the fungal cell wall and binds directly to ergosterol in the fungal plasma membrane; (B.1–B.2) updated model, showing that intact LAmB vesicles traverse fungal cell wall and reach the plasma membrane, where AmB is subsequently transferred to ergosterol without extensive release into the extracellular environment; adapted from Soo Hoo (2017).

Several theories have been proposed to explain the antifungal mechanism of LAmB. Initially, it was suggested that AmB, with its affinity for ergosterol being ten times greater than that for liposomal cholesterol, would detach from the liposome bilayer upon binding to the cell wall. This would cause liposomal breakdown near the fungal cell membrane, allowing free AmB to pass through and interact with ergosterol in the membrane (Adler-Moore et al., 2019). However, recent studies have revealed that LAmB transits intact through the cell walls of *C. albicans* and *Cryptococcus neoformans*, despite its size (60–80 nm) exceeding the theoretical pore size of the cell walls. These findings suggest that the cell walls of these fungi possess deformable, viscoelastic properties that allow vesicular trafficking across the wall. Eventually, liposomes release AmB through membrane diffusion and bind to ergosterol in the fungal cell membrane. These findings clarify why LAmB has low toxicity; it delivers its cargo directly to the cell membrane, bypassing the significant release of free AmB into the surrounding aqueous environment during systemic circulation (Walker et al., 2018). These mechanistic pathways are summarised in Fig. 3, which depicts both the traditional model of AmB release at the cell wall surface and the recent evidence indicating that intact LAmB can traverse the fungal cell wall before binding to ergosterol.

In summary, liposomal nanoformulations, particularly those less than 100 nm in size, can diffuse rapidly across the fungal cell wall intact and release AmB directly at the membrane. Owing to the high affinity of ergosterol in the fungal cell membrane compared to cholesterol in human cells, AmB can bind preferentially and disrupt the fungal membrane. This unique combination of fungal cell wall penetration and ergosterol targeting, offered by AmBisome®, can deliver a 10-fold higher dose than AmB in deoxycholate formulation without causing significant nephrotoxicity, indicating AmBisome's high safety, tolerability, and efficacy in clinical settings.

LAmB has demonstrated superior safety at significantly higher doses of AmB, ranging from 10 to 20 mg/kg/day, when it is administered in various animal models (including mice and rabbits) (Lestner et al., 2017). These findings led to later clinical trials in which high-dose LAmB was used to treat invasive fungal infections (Gu et al., 2023). A phase I/II study evaluating the safety, tolerability, and pharmacokinetics of high-dose LAmB revealed that doses up to 15 mg/kg/day were well tolerated, with no nephrotoxicity or infusion-related toxicity observed, and showed equivalent efficacy in treating aspergillosis and other filamentous fungal infections compared with the conventional AmB deoxycholate formulation (Walsh et al., 2001). In patients treated with LAmB, tissue concentrations are typically highest in the liver and spleen, with significantly lower levels observed in the kidneys and lungs (Hamill, 2013). LAmB has usually been recommended for febrile or neutropenic patients resulting from fungal infections such as aspergillosis, candidiasis, and cryptococcosis and those that are refractory to AmB deoxycholate (Seibel Nita et al., 2017). A phase III randomised controlled trial conducted by Jarvis et al. (Jarvis et al., 2022) demonstrated that a single high dose (10 mg/kg) of LAmB, given on an optimised oral backbone of fluconazole and flucytosine for two weeks, is effective in treating cryptococcal meningitis, significantly improving patient outcomes compared with conventional treatment regimens. Based on these findings, the WHO has endorsed a regimen based on a single dose of LAmB at 10 mg/kg as the preferred treatment approach for patients with cryptococcal meningitis (WHO, 2022a).

Owing to its favourable safety profile, ability to deliver high doses with minimal systemic toxicity, and controlled membrane diffusion-based release, LAmB has been increasingly investigated for direct pulmonary administration, both as prophylaxis and as a treatment for pulmonary aspergillosis (Adler-Moore and Proffitt, 2002; Stone et al., 2016). Compared with systemic antifungal therapies, nebulised LAmB delivery offers several clinical advantages, including excellent drug tolerance, effective local concentrations at infection sites achieved at lower doses, minimal systemic absorption, and fewer drug interactions (Akinosoglou et al., 2024; Godet et al., 2015). A randomised, placebo-controlled trial involving 271 lung transplant recipients with prolonged neutropenia tested the effects of inhaling 12.5 mg LAmB twice weekly. The results showed that prophylactic inhalation of liposomal amphotericin B significantly decreased the occurrence of invasive pulmonary aspergillosis in high-risk patients (Rijnders et al., 2008). The small size and negative charge of LAmB liposomes shift the usual macrophage response from a proinflammatory to an anti-inflammatory cytokine profile, thereby reducing the upregulation of proinflammatory cytokines and mitigating infusion-related adverse reactions (Arning et al., 1995; Bellocchio et al., 2005; Simitsopoulou et al., 2005).

Overall, the LAmB formulation has been shown to offer a safer option than conventional AmB formulations for all the clinical conditions investigated, particularly in reducing nephrotoxicity and providing more renal protection (Falci et al., 2015). Additionally, there seems to be a trend toward fewer infusion-related reactions, particularly when LAmB is used to treat chronic conditions. Notably, LAmB stands out as arguably the most successful lipid-based formulation to date, establishing itself as the gold standard in AmB-related clinical use (Lawrence et al., 2022). Moreover, despite their relatively high cost, LAmB is cost-effective in specific clinical settings because it results in a short hospital stay, few adverse events, and good overall patient outcomes [119]. Nevertheless, the substantially higher per-unit cost of lipid-based formulations remains a considerable barrier in many resource-limited settings, where conventional AmB continues to be used, primarily owing to its lower initial cost (Grazziotin et al., 2018): LAmB is almost 100 times more expensive than conventional AmB in private healthcare settings (Faustino and Pinheiro, 2020; Steimbach et al., 2017; Vyas and Gupta, 2006).

## 3 Antibacterial drug Amikacin

Despite being among the oldest antibacterial agents, aminoglycosides have continued to attract significant interest in antibacterial chemotherapy (Bush, 2012; Raut et al., 2022). When they were first discovered, aminoglycosides demonstrated strong intrinsic efficacy against gram-negative and selected gram-positive bacterial infections, as well as *Pseudomonas* spp. and *mycobacteria* (Becker and Cooper, 2013; Raut et al., 2022). As these antibiotics have become more commonly used in clinical practice, resistance has emerged, and toxicological risks, especially ototoxicity and nephrotoxicity, have become increasingly apparent (Becker and Cooper, 2013). During the early 1970s, to increase the therapeutic efficacy and reduce the toxicity associated with first-generation (natural) aminoglycoside antibiotics, second-generation aminoglycosides (semisynthetic derivatives), including AMK, were introduced (Raut et al., 2022).

**Fig. 4:**
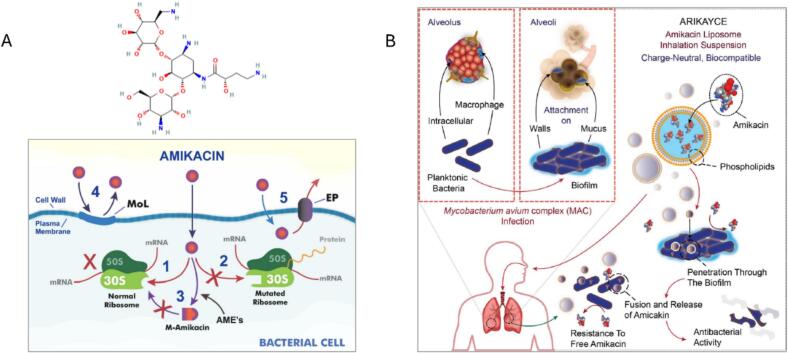
Various proposed mechanisms of bacterial resistance toward amikacin (A) and the use of liposomal amikacin for overcoming some of the resistance mechanisms; an example of Arikayce® penetrating the biofilm and providing sustained release of the drug through fusion onto the cell membrane of the bacterial cells (B); adapted from Maxwell et al. (2021).

AMK, a semisynthetic derivative of kanamycin, was developed in 1972 and has become one of the most commonly used aminoglycoside antibiotics (Maxwell et al., 2021) (Fig. 4). AMK, a white solid crystalline powder generally used as a sulfate salt, is freely soluble in water with a solubility of 185 g/L at 25 °C and belongs to the class III drugs of the BCS (Fatima et al., 2018; Raut et al., 2022). Owing to its polarity, AMK is poorly absorbed via oral administration and has poor intestinal permeability. Instead, it is administered intravenously, intramuscularly, or through nebulisation (Hassan et al., 2018; Olivier et al., 2014; Ramirez and Tolmasky, 2017; Routledge and Hutchings, 2013). The polycationic nature of AMK facilitates its interaction with negatively charged components on the bacterial surface, such as phospholipids and lipopolysaccharides. This interaction increases the permeability of the cytoplasmic cell membrane in susceptible bacteria, enhancing AMK uptake into the periplasmic space, where it binds to bacterial ribosomes and exerts its action (Maxwell et al., 2021). Like all aminoglycoside antibiotics, AMK inhibits protein synthesis by binding to the 30S ribosomal subunits of susceptible bacteria. This binding impairs the ribosome's proofreading ability, increasing mistranslation and disrupting protein synthesis, which leads to the production of toxic or nonfunctional peptides (Ramirez and Tolmasky, 2017). Various mechanisms have been proposed by which resistance to AMK arises, including alterations in the binding site and the action of aminoglycoside-modifying enzymes (Fig. 3). Additionally, AMK entry into the cell may be prevented by modifying membrane lipids and by enhancing the activity of bacterial efflux pumps [166].

AMK can be administered as monotherapy and in combination with other antibiotics to treat various bacterial infections, including bloodstream infections, UTIs, and meningitis. Additionally, it is extensively used to treat chronic respiratory airway infections (Caminero et al., 2010; Daley et al., 2020; Dellagrammaticas et al., 2000; Raaijmakers et al., 2021). AMK is a concentration-dependent antibiotic with bactericidal activity (Maxwell et al., 2021). The standard regimen for AMK involves once-daily administration at a therapeutic dose of 15 mg/kg to achieve effective minimum inhibitory concentrations for patients in general wards and ICUs (De Winter et al., 2018). Despite its efficacy, the short half-life of AMK necessitates frequent administration at higher doses, potentially leading to progressive adverse effects, including ototoxicity, vestibulotoxicity, and nephrotoxicity, which are common toxicities associated with aminoglycosides (Aznar et al., 2019; Fatima et al., 2018). Given its narrow therapeutic index and significant potential for toxicity, AMK is typically administered via slow intravenous infusion (Maxwell et al., 2022; Ramirez and Tolmasky, 2017). Overall, the poor oral bioavailability, short half-life, and severe toxicity of AMK necessitate frequent intravenous dosing, accompanied by careful monitoring of drug concentrations to maintain plasma levels within the narrow therapeutic range (Fielding et al., 1999). In response to these challenges, MiKasome®, a sterile intravenous formulation of AMK encapsulated in liposomes, was developed by NeXstar Pharmaceuticals to evaluate potential improvements in the safety and efficacy of AMK using small, low-clearance liposomes (Drulis-Kawa and Dorotkiewicz-Jach, 2010; Fielding et al., 1998b). MiKasome® is a formulation of small unilamellar liposomes (with a diameter < 100 nm) consisting of AMK base, HSPC, DSPG, and cholesterol in sucrose/succinate buffer (pH 6). The AMK concentration in the formulation was approximately 10 mg/mL (Fielding et al., 1998b). MiKasome® has completed phase 2 clinical trials for the eradication of mycobacteria. Whitehead et al. (1998) conducted a study of patients with multidrug-resistant tuberculosis and found that MiKasome® was well tolerated and achieved 30-fold higher accumulation in serum and sputum than its free-drug counterpart, with a significantly longer half-life of 180 h compared with 6 h for conventional AMK. Importantly, MiKasome® demonstrated lower toxicity, with no renal or significant hearing disorders observed, in contrast to the free drug. However, the formulation did not eradicate *Mycobacterium tuberculosis* from sputum or reduce drug sensitivity, highlighting the need for further efficacy and dosing studies. Following Gilead Sciences' acquisition of NeXstar Pharmaceuticals in 1999, additional clinical trials of this formulation were postponed due to insufficient clinical evidence, resulting in the discontinuation of product development (Drulis-Kawa and Dorotkiewicz-Jach, 2010; Maxwell et al., 2021).

Despite the failure to develop a liposomal formulation of AMK for intravenous administration, this class of drugs have several characteristics that render them suitable for pulmonary administration. First, as concentration-dependent antibiotics, aminoglycosides can achieve relatively high local drug concentrations in the lungs via inhalation, potentially increasing their bactericidal activity. Second, intravenous administration of high doses of aminoglycosides can be associated with systemic toxicity while only achieving minimal pulmonary accumulation. In contrast, AMK delivered via inhalation has consistently shown intrapulmonary concentrations that are 20 times higher than those obtained via the intravenous route (Elman et al., 2002; Wenzler et al., 2016b). However, due to their hydrophilic nature and low molecular weight, inhaled aminoglycosides such as AMK are rapidly absorbed systemically and quickly cleared from the lungs, resulting in a short pulmonary half-life (Meers et al., 2008; Stockmann et al., 2015). This rapid clearance reduces the duration for which drug concentrations remain above the effective minimum inhibitory concentration at the infection site (Meers et al., 2008). Liposomal formulations have been developed to overcome rapid drug clearance and systemic absorption, thereby prolonging the pulmonary retention of hydrophilic aminoglycosides. These formulations provide sustained drug release at the site of infection, prolonging bacterial exposure to the antibiotic and enhancing bacterial eradication. Compared with conventional therapies, liposomal-encapsulated antibiotics have shown improved delivery and efficacy against biofilm-associated bacteria (Alhariri et al., 2017; Meers et al., 2008; Panthi et al., 2024). Additionally, liposomal antibiotics are efficiently taken up by macrophages, increasing the intracellular drug residence time and enhancing the targeting of intracellular pathogens (Kelly et al., 2011; Wenzler et al., 2016a). These beneficial attributes make liposomes highly suitable carriers for inhaled antibiotic delivery to manage pulmonary infections (Justo and Moraes, 2005; Vyas et al., 2004).

### 3.1 Liposomal AMK inhalation suspension (Arikayce®)

Arikayce® (AMK liposome inhalation suspension [ALIS]) is an AMK sulfate encapsulated in liposomes for delivery to the lungs via aerosol nebulisation (Khan and Chaudary, 2020). This formulation received FDA approval in 2018, followed by EMA approval in 2020 (Liu et al., 2022). Arikayce**®** comprises dipalmitoylphosphatidylcholine (DPPC) and cholesterol liposome at a 2:1 weight ratio with a total lipid-to-drug weight ratio in the range of 0.60–0.79 (70 mg/mL AMK sulfate and 47 mg/mL lipids) (Shirley, 2019; Weers et al., 2009). Lipids used in Arikayce® are structurally similar to pulmonary surfactants, thus facilitating their clearance from the lungs via common pathways (Waters and Ratjen, 2014). These lipids also provide a high degree of membrane stability and reduce drug leakage owing to their high phase transition temperature. This ensures efficient nebulised delivery and maintains high concentrations of AMK in the lungs (Meers et al., 2008). It is provided as a sterile, white, milky, aqueous liposomal suspension in a vial containing 590 mg of AMK (Zhang and Hill, 2021). Arikayce**®** liposomes are neutral in charge and relatively small, measuring 300 nm in diameter. The neutral charge of liposomes can shield polycationic AMK from negatively charged sputum components, enhancing its stability in the lung. Additionally, these liposomes can penetrate the sputum and dense biofilm of bacteria, such as *P. aeruginosa* and nontuberculous mycobacteria (NTM) (Fig. 3) (Meers et al., 2008; Waters and Ratjen, 2014; Zhang et al., 2018). High drug loading in Arikayce**®** enables effective delivery of AMK to the patient's lungs in approximately 14 min via a product-specific eFlow® nebuliser (Lamira™ nebuliser system) (FDA, 2018a). Despite the high aqueous solubility of AMK sulfate, over 98 % of the AMK in Arikayce® is encapsulated within liposomes. The high encapsulation efficiency results from the drug's limited solubility in ethanol during the in-line infusion manufacturing process, which causes the formation of a transient coacervate phase that becomes trapped within the liposomal aqueous core, while its multicationic nature limits diffusion across the neutral DPPC/cholesterol bilayer (Boni et al., 2014; EMA, 2020b). However, 30 % is released during nebulisation. Aerosolised liposomes maintain a mean size of ∼300 nm after delivery to the lung. The Lamira™ nebuliser system produces droplets within the respirable range (1–5 μm), with 67 % of aerosol droplets being less than 5.0 μm, resulting in 43 % of the nominal liposome dose being deposited in the lungs (Olivier et al., 2017; Waters and Ratjen, 2014; Zhang and Hill, 2021). Studies have also demonstrated that ALIS is homogeneously distributed throughout various lung lobes and effectively reaches the distal airways (Malinin et al., 2016; Meers et al., 2008).

Preclinical studies in rat lungs have shown that Arikayce® can penetrate *P. aeruginosa* biofilms and infect mucus, delivering AMK in a slow and sustained manner (Meers et al., 2008). To evaluate the deposition and clearance of Arikayce®, a study using three healthy volunteers and gamma scintigraphy found that a single 120 mg dose of radiolabelled liposomal AMK resulted in prolonged retention of drug-loaded liposomes in the lungs but was well tolerated, with no significant clinical changes (Weers et al., 2009). Given its enhanced lung deposition, Arikayce® has proven to be an attractive therapeutic choice for cystic fibrosis (CF) patients with *P. aeruginosa* lung infections, outperforming free AMK (Ehsan and Clancy, 2015). Initial pharmacokinetic and pharmacodynamic evaluations in 24 CF patients confirmed its antibacterial efficacy at 500 mg/day, supporting this dosage for further clinical trials (Okusanya et al., 2009). A phase II clinical trial evaluated the safety and efficacy of a 28-day regimen of once-daily Arikayce® in CF patients with chronic *P. aeruginosa* infections. This randomised, placebo-controlled study examined the pharmacokinetics and efficacy of four Arikayce® doses. The results showed that the 280 mg and 560 mg doses improved lung function, as evidenced by increased forced expiratory volume (FEV1), with the 560 mg dose sustaining improvements for 56 days and significantly reducing sputum density. Arikayce® was well tolerated, and adverse effects were consistent with those observed in CF-related lung disease, demonstrating dose-dependent therapeutic effects (Clancy et al., 2013). Extensive preclinical studies confirmed that Arikayce® is concentrated in alveolar macrophages without impairing their killing function or cytokine signalling (Malinin et al., 2016). Macrophages in the lung efficiently accumulate AMK during treatment and almost eliminate it during recovery, thereby preventing an inflammatory response or functional compromise. These findings highlight the potential of Arikayce® to treat intracellular pathogens such as NTM without causing systemic toxicity, benefiting both CF and non-CF patients (Ehsan and Clancy, 2015).

Managing pulmonary NTM infections, such as *Mycobacterium avium complex* (MAC), in CF patients is particularly challenging, often requiring prolonged treatment with intravenous AMK and other antibiotics for months or even years (Waters and Ratjen, 2016). ALIS represents a promising and alternative delivery method for NTM-induced pulmonary infections. Preclinical studies in rat models demonstrated that aerosolised delivery of liposome-encapsulated AMK resulted in lung AMK levels up to 274 times higher than those achieved with intravenous AMK. Confocal microscopy further confirmed that liposomal AMK effectively penetrated and eliminated biofilms. Free inhaled AMK is significantly less effective than its liposome-encapsulated counterpart, confirming the therapeutic superiority of liposomal delivery (Zhang et al., 2018). Notably, systemic exposure to AMK in the serum and urine following once-daily Arikayce® treatment in patients with refractory NTM lung disease was significantly lower than that previously reported for using the native AMK (Rubino et al., 2021). On the other hand, post-dose sputum levels of AMK are substantially higher than its C_max_ in serum following Arikayce® administration (FDA, 2018a). When Arikayce® was combined with guideline-based therapy, 29 % of NTM infection cases were cleared, a superior outcome compared with guideline-based therapy alone (Griffith et al., 2018). Consequently, in late 2018, the FDA granted accelerated approval for Arikayce® to treat MAC-related lung disease in adults with limited treatment options. Arikayce® remains the only drug approved by the FDA, EMA, and Japan for treating MAC-related lung disease when no other treatment alternatives are available, another success for liposomal antimicrobials (EMA, 2020a; FDA, 2018a).

Arikayce® inhalation indeed reduces AMK systemic toxicity, including ototoxicity and nephrotoxicity, which are typically associated with intravenous administration. However, its use has been linked to respiratory toxicity, including dyspnoea, cough, haemoptysis, and dysphonia (Griffith et al., 2018). Overall, inhaled liposomal AMK represents a significant advancement in the development of new antimicrobials for treating difficult-to-treat pulmonary infections caused by multidrug-resistant organisms (Waters and Ratjen, 2014). In addition to the success of marketed products such as AmBisome® and Arikayce®, further research highlights the potential of liposomes as antimicrobial carriers. Their versatility in carrying payloads, penetrating the complex interfaces of biohydrogels (mucosal barriers) and biofilms, and tailoring their pharmacological distribution and modulating their release profiles offers distinct advantages over conventional approaches, particularly in tackling AMR-associated infections.

## 4 Strategies using liposomes to overcome antimicrobial resistance

Building on the proven success of marketed liposomal products, research has demonstrated the broader potential of liposomes as antimicrobial nanocarriers. By encapsulating antimicrobial agents, liposomes enhance drug stability (Oh et al., 1995) and safety (Li et al., 2017), offering improved pharmacokinetic and pharmacodynamic profiles (Fielding et al., 1998a; Stone et al., 2016). They prolong the circulation time of the drug in the bloodstream (Bakker-Woudenberg et al., 2005; Bakker-Woudenberg et al., 1995), enable site-specific delivery (Marier et al., 2003), and address resistance mechanisms by penetrating the barriers in the microenvironment (Li et al., 2008) and modulating drug release (Maxwell et al., 2021). These characteristics position liposomes as versatile tools to overcome challenges associated with AMR-related infections, paving the way for new and exciting AMR therapeutic strategies.

### 4.1 Encapsulation, controlled release and resistance evasion

Pathogens have developed various resistance mechanisms that compromise the efficacy of antibiotics, including enzymatic degradation (e.g., β-lactamases) and chemical modification (e.g., acetyltransferases), rendering certain antibiotics inactive (Ghosh and De, 2023). Encapsulation within liposomal vesicles helps overcome these challenges by shielding antibiotics not only from bacterial enzymatic degradation but also from host-induced hydrolysis, chemical instability, and immune system deactivation (Allen, 1998; Eleraky et al., 2020). Research has demonstrated that encapsulating piperacillin in phosphatidylcholine–cholesterol liposomes protects it from degradation by staphylococcal β-lactamases, thereby maintaining its antibacterial efficacy (Nacucchio et al., 1985). Encapsulation of antibiotics in liposomes enables controlled drug release, reducing premature drug loss and the frequency of administration. The release profile is influenced by the liposomal composition, with stimuli-responsive liposomes, such as temperature- or pH-sensitive formulations, offering targeted drug delivery by disintegrating under specific conditions (Alzahrani et al., 2022; Drulis-Kawa and Dorotkiewicz-Jach, 2010; Ferreira et al., 2021b). pH-sensitive liposomes remain stable in circulation but become leaky in a pH-altered environment, enabling controlled drug release. Vesicles containing dioleoyl phosphatidylethanolamine (DOPE) lipids have been used to target intracellular pathogens such as *Salmonella* spp. Gentamicin encapsulated in these pH-sensitive liposomes demonstrated greater efficacy against vacuole-resident *Salmonella Typhimurium* than DPPC-containing liposomal formulations did (Cordeiro et al., 2000; Lutwyche et al., 1998).

Another targeted strategy for controlling antibiotic release and enhancing delivery within biofilms involves stimulus-responsive liposomes that release their payload in response to bacterial enzymes (Ding et al., 2022). For example, phospholipase A2 (PLA2), secreted by *Helicobacter pylori*, hydrolyses membrane phospholipids, thereby compromising mucosal integrity and facilitating bacterial survival. Liposomes formulated with PLA2-sensitive lipids can degrade in the presence of *H. pylori*, triggering the controlled release of encapsulated antibiotics at the infection site (Thamphiwatana et al., 2014).

In addition to protecting antimicrobials from degradation, liposomes can be used to evade microbial resistance mechanisms by enhancing membrane interactions and counteracting efflux pumps. The multidrug-resistant efflux pump is another bacterial defence mechanism that lowers the intracellular concentration of antimicrobial agents to sublethal levels, allowing bacteria to survive their bactericidal effects. The potential to use liposomes to resist efflux pump transportation of antimicrobials back out of microorganisms has been exemplified by the use of liposomal ciprofloxacin against MRSA (Faraag et al., 2022). In this study, liposomal ciprofloxacin triggers a less potent stress response than the free drug, consistent with reduced efflux pump expression.

### 4.2 Biofilm penetration and eradication

Microbial biofilms, which are formed by both bacteria and fungi, constitute a significant mechanism of antimicrobial resistance. These biofilms consist of structured communities of microbial cells embedded within a self-produced extracellular polymeric substance (EPS) matrix (Ferreira et al., 2021b; Liu et al., 2019; Mitchell et al., 2016). The EPS matrix is a complex, three-dimensional network composed primarily of polysaccharides, proteins, extracellular DNA, and lipids that collectively provide structural integrity, adhesion properties, and protection for microbial communities. This matrix acts as a physical barrier that entraps microbial cells and increases the thickness of the aqueous boundary layers outside the cell wall, thereby hindering the diffusion of antimicrobial drugs (Shree et al., 2023). Additionally, the tiny pores within this polymeric network hinder the passage of larger drug molecules, further reducing antimicrobial efficacy (Flemming and Wingender, 2010; Gilbert et al., 2002). As a result, the antimicrobial dose required to eliminate biofilms can be up to 1000 times greater than that needed to eradicate planktonic cells (Mah and O'Toole, 2001). In such cases, high concentrations of free antimicrobials may be necessary to achieve therapeutic effects due to low target specificity; however, this approach increases the risk of unintended tissue damage and toxicity (Forier et al., 2014). Therefore, a drug delivery system capable of penetrating biofilms is often needed to increase antimicrobial efficacy and circumvent such resistance mechanisms (Wang, 2021).

Liposomes have been extensively studied for their potential to penetrate and manage infectious biofilms (Dos Santos Ramos et al., 2018). The physicochemical properties of liposomes have been shown to affect their effectiveness in preventing and eradicating biofilms. Key features, such as particle size, particle size distribution, lipid composition, and surface characteristics, significantly influence their effectiveness in delivering antimicrobial agents to biofilm-associated infections (Rukavina and Vanić, 2016). The particle size of nanocarriers is a key factor, as it determines their ability to traverse biofilms and should remain below the dimensions of water-filled channels. Defining the sizes of these channels is challenging, as the distinction among water-filled channels, pores, and voids within biofilms is not well established and may depend on bacterial species. Generally, their dimensions range from ∼10 nm to several μm, with nanometre-scale structures corresponding to narrow aqueous connections between bacterial aggregates, and micrometre-scale spaces representing larger pores or void regions. Nanocarriers with diameters of 5–200 nm are considered optimal for controlling biofilm-associated infections, whereas particles larger than 500 nm are unlikely to penetrate biofilms efficiently (Liu et al., 2019). Several studies have evaluated the impact of liposomal surface charge on drug delivery, drug uptake, and biofilm eradication. Drulis-Kawa et al. (2009) investigated various anionic and cationic liposomal formulations for meropenem delivery in an in vitro model of *P. aeruginosa* biofilms. Their findings showed that cationic liposomes were more effective, likely because of ionic interactions and enhanced fusion with the bacterial cell membrane. Alhajlan et al. (2013) examined the efficacy and safety of liposomal clarithromycin for treating *P. aeruginosa* biofilm infections. The drug was encapsulated in neutral (DPPC/Chol), anionic (DPPC/Chol/dicetylphosphate), and cationic (DPPC/Chol/dimethyldioctadecylammonium bromide (DDAB)) liposomes, all approximately 200 nm in diameter. Microbiological evaluations confirmed that both anionic and cationic formulations successfully eradicated *P. aeruginosa* biofilms, with the cationic liposomes exhibiting the most pronounced effect. While bacterial biofilms pose significant challenges to antibiotic delivery, fungal biofilms also pose barriers to effective antifungal therapy, limiting drug penetration and contributing to the development of resistance. The development of *Candida* spp. biofilms present an additional clinical challenge by further enhancing antifungal resistance and enabling evasion of host immune defences. Compared with conventional deoxycholate formulations, liposomal formulations have been shown to mitigate these issues and offer superior efficacy in eradicating biofilm-associated *Candida* spp. (Rodrigues and Henriques, 2017). More recent studies also suggest that liposomal formulations of other antifungals, such as anidulafungin, are more effective at disrupting mature *Candida albicans* biofilms than the free drug alone. This behaviour has been attributed to the presence of hexosamines, positively charged sugars in fungal biofilms, which may interact with negatively charged liposomes, thereby enhancing nanoparticle penetration and transport through the biofilm matrix (Sardi et al., 2013; Vera-González et al., 2020). Although the *C. albicans* cell wall is overall negatively charged, its composition changes markedly during morphologic transition, with chitin – a cationic sugar – increasing approximately three-fold in hyphal cells compared with yeast, while mannan levels decrease. These molecular changes account for the presence of cationic sugar domains in hyphal biofilms and support electrostatic interactions between anionic liposomes and chitin-rich regions, thereby enhancing antifungal delivery and biofilm disruption (LaMastro et al., 2023; Wang et al., 2015).

### 4.3 Targeting microorganisms and interactions with biofilms

The nature and structural characteristics of liposomes resemble those of host cells and certain microorganisms, including bacteria, enabling effective interactions between these carriers and target cells (de Leeuw et al., 2009; Nicolosi et al., 2010). These interactions are generally categorised into four processes: adsorption, endocytosis, lipid exchange, and fusion (Düzgüneş and Nir, 1999). Fusion, in particular, facilitates more efficient intracellular drug delivery by allowing direct transfer of therapeutic agents into cells (Goryacheva et al., 2005; Watabe et al., 1999). Fusogenic liposomes, with their bilayers exhibiting a liquid crystalline phase, have demonstrated an enhanced ability to interact with cell membranes, favouring the reciprocal mixing and release of vesicle content inside cells (Nicolosi et al., 2010; Scriboni et al., 2019). When formulated with fusogenic lipids, e.g., DOPE, these liposomes further increase membrane fluidity, promoting bacterial wall destabilisation and facilitating drug release inside resistant pathogens (Atashbeyk et al., 2014; Huang et al., 2011). This mechanism is effective against *P. aeruginosa*, achieving antibacterial effects even at sub-MIC concentrations (Sachetelli et al., 2000). *P. aeruginosa* resistance is primarily attributed to its low outer membrane permeability (Hancock and Brinkman, 2002). The incorporation of fusogenic properties into liposomal antibiotics offers a potential strategy to bypass this resistance mechanism by enhancing drug targeting and intracellular delivery (Drulis-Kawa and Dorotkiewicz-Jach, 2010). For example, fusogenic liposomes have been used to increase the delivery of vancomycin, a glycopeptide antibiotic, by bypassing the outer membrane barrier. This approach has been shown to overcome resistance in several clinically relevant gram-negative pathogens (Nicolosi et al., 2010). The likely reason is that vancomycin has the highest efficacy when it binds to murein monomers within the cytoplasm before they are incorporated into the peptidoglycan layer (Hiramatsu, 2001).

Modification of the liposomal surface can improve microbial binding and drug delivery. For example, liposomes conjugated with wheat germ agglutinin (WGA) facilitated the transport of clarithromycin into MRSA biofilms. As WGA binds to bacterial membrane components, it promotes intracellular drug release (Meng et al., 2016). This has also been observed in the treatment of the fungal pathogen *C. neoformans*, where liposomes coated with dectin, a host receptor, can exhibit a stronger binding affinity to yeast than conventional liposomes. Furthermore, compared with traditional AmB-loaded liposomes, dectin-coated AmB-loaded liposomes significantly reduce fungal burden in the lungs, brain, kidneys, liver, and spleen in a systemic *Cryptococcus* model, thereby prolonging survival in mice (Pham et al., 2024). To counteract the sequestering effects of the biofilm extracellular polymeric matrix, the incorporation of biofilm-disrupting agents, such as DNases and proteinases, into liposomes has been shown to degrade the architecture of *Cutibacterium acnes* biofilms (Fang et al., 2021).

### 4.4 Reduced toxicity and side effects

As previously discussed, managing infections with persistent biofilms often requires high doses of antimicrobial agents, which can cause adverse effects, including allergic reactions and off-target toxicity, thereby promoting the development of antibiotic tolerance and resistance (Kalelkar et al., 2022; Liu et al., 2020a). Liposomal formulations, composed of biocompatible, biodegradable, and relatively nontoxic lipids, can mitigate these toxic effects by encapsulating antimicrobial agents (Allen, 1998; Rukavina and Vanić, 2016). Liposomes improve the safety of hydrophobic antimicrobial agents by encapsulating them, shielding the drug from the aqueous environment, and enhancing its stability in aqueous media, thereby preventing in vivo precipitation. A key factor in drug toxicity is nonspecific biodistribution. Liposomes reduce this ability by enabling targeted delivery, either passively or actively, directing the drug to the intended site of action and reducing off-target effects (Lim et al., 2012). Furthermore, liposomes provide a sustained-release profile that reduces toxicity, the need for frequent dosing, and fluctuations in plasma drug levels. Ideally, targeted systems should prevent drug release during circulation, thereby ensuring maximum accumulation at the target site (Hamidi et al., 2006; Lim et al., 2012). Severe bacterial infections often require high doses of aminoglycosides (AMK, gentamicin, and tobramycin), which are associated with severe toxic effects, including nephrotoxicity and ototoxicity (Nagai and Takano, 2004). To improve their safety profile and enhance their therapeutic index, aminoglycosides have been encapsulated in liposomes. Compared with both rigid liposomal tobramycin and the free drug, fluidosomes®, a fluid liposomal tobramycin formulation, exhibited superior lung-targeted drug retention but significantly reduced renal exposure. Such localised delivery can minimise systemic toxicity while maintaining high antibacterial efficacy (Sachetelli et al., 1999). Similarly, liposomal encapsulation has shown considerable promise in reducing the systemic toxicity of antifungal agents. Compared with free nystatin, Nyotran® (a multilamellar liposomal formulation of nystatin) demonstrated significantly reduced hemolytic activity, lower renal toxicity, and enhanced overall tolerability. This favourable safety profile enabled the administration of higher doses, resulting in improved antifungal efficacy against invasive candidiasis and aspergillosis (Mehta et al., 1987; Sousa et al., 2023).

## 5 Structure of interest for new liposomal antimicrobials

When designing liposomal drug delivery systems for AMR applications, multiple factors must be considered. The chemical composition and structural properties of liposomes should be optimised to ensure adequate performance in clinically relevant microenvironments, with these relationships thoroughly explored during development. A summary of clinically approved liposomal nanomedicines for oncology and other diseases may inform the design of liposomal antimicrobials (Table 2). Central to the liposomal composition are phospholipids, which are composed of a hydrophilic phosphate head and two hydrophobic fatty acyl tails, both of which are linked by an alcohol moiety, i.e., glycerol (Pinot et al., 2014). Due to their amphiphilic properties, phospholipids tend to self-assemble in aqueous environments, driven by the strong interaction between the hydrophilic head and water molecules, as well as the inherent tendency of the hydrophobic tail to repel water (Baccile et al., 2021). Phospholipids are generally classified into two types: natural and synthetic. The natural phospholipids used in liposome manufacturing can be obtained from plant sources (Antimisiaris et al., 2008; van Hoogevest and Wendel, 2014). Modifications to the head groups, fatty acyl chains, and alcohols of natural phospholipids, introduced via tailor-made chemical processes, yield a range of synthetic lipids (Guimarães et al., 2021). Synthetic phospholipids are commercially manufactured and share biological and structural similarities with natural phospholipids. Although not endogenous, synthetic phospholipids offer excellent stability, purity, and biocompatibility, making them suitable for parenteral liposomal formulations (Large et al., 2021; Li et al., 2015). Equally, natural phospholipids are typically preferred because of their cost-effectiveness, large-scale availability, environmentally friendly production, and ease of regulatory approval (van Hoogevest and Wendel, 2014).

**Table 2:** Lists of liposomal products approved by the FDA and EMA.*.

Name	API	Lipids (molar ratio)	Indication	Approval
DaunoXome®	Daunorubicin	DSPC: chol 2:1	AIDS-related Kaposi's sarcoma	EU (1997) US (1996)
Doxil®	Doxorubicin	HSPC:chol:DSPE-PEG 56:39:5	Ovarian, breast cancer, Kaposi's sarcoma	EU (1996) US (1995)
DepoCyt®	Cytarabine	DOPC:DPPG:chol:triolein 5:1:9:1	Neoplastic meningitis	US (1999)
Exparel®	Bupivacaine	DEPC:DPPG:chol: tricaprylin 9:1:10:4	Pain management	US (2011)
Epaxal®	Inactivated hepatitis A virus	DOPC: DOPE 3:1	Hepatitis A	EU (1999)
Inflexal V	Influenza virus surface antigens	DOPC: DOPE 3:1	Influenza	EU (2000)
Lipodox®	Doxorubicin	HSPC:chol:DSPE-PEG 56:39:5	Ovarian, breast cancer, and Kaposi's sarcoma	US (2013)
Marqibo®	Vincristine	SPH: chol 3:2	Leukaemia	US (2012)
Mepact®	Mifamurtide	DOPC: DOPS 3:7	osteosarcoma	EU (2009)
Mosquirix®	RTS, S antigen-based vaccine	DOPC:chol:MPL-A:QS-21 2:1:0.05:0.04	Malaria	EU (2015)
Myocet®	Doxorubicin	EPC: chol 11:9	Metastatic breast cancer	EU (2000)
Onivyde®	Irinotecan	DSPC:chol:DSPE-PEG 3:2:0.015	Pancreatic adenocarcinoma	US (2015) EU (2016)
Shingrix®	Recombinant varicella-zoster virus glycoprotein E	EPG: DMPC 3:5	Shingles and postherpetic neuralgia	US (2017) EU (2018)
Vyxeos®	Daunorubicin/cytarabine	DSPC:DSPG:chol 7:2:1	Leukaemia	US (2017) EU (2018)

*Abbreviations: chol: cholesterol, DEPC: 1,2-dierucoyl phosphatidylcholine, DMPC: dimyristoyl phosphatidylcholine, DMPG: dimyristoyl phosphatidylglycerol, DOPC: dioleoyl phosphatidylcholine, DOPE: dioleoyl phosphatidylethanolamine, DOPS: dioleoyl phospatidylserine, DPPC: dipalmitoyl phosphatidylcholine, DPPG: dipalmitoyl phosphatidylglycerol, DSPC: distearoyl phosphatidylcholine, DSPG: distearoyl phosphatidylglycerol, DSPE-PEG: distearoyl phosphatidylcholine polyethylene glycol, EPC: egg phosphatidylcholine, EPG: egg phosphatidylglycerol, HSPC: hydrogenated soy phosphatidylcholine, SPH: sphingomyelin, MPL-A: monophosphoryl lipid A, QS-21: *Quillaja saponaria* fraction 21.

The properties of liposomes are often strongly influenced by the characteristics of lipids and their compositions. Depending on the nature of the lipid head, liposomes can acquire positive, negative, or neutral charges (Lombardo et al., 2016). Examples of anionic lipids include dioleoyl phosphatidylglycerol (DOPG) and dimyristoyl phosphatidylglycerol (DMPG), whereas cationic lipids include dioleoyl trimethylammonium-propane (DOTAP) and DDAB. Adding anionic or cationic lipids to liposomes improves colloidal stability by creating electrostatic repulsion between vesicles, which helps prevent aggregation (Lombardo and Kiselev, 2022). While cationic liposomes offer enhanced electrostatic attraction to negatively charged bacterial membranes, significantly improving targeted antibiotic delivery (Singh et al., 2024). Anionic liposomes demonstrate superior encapsulation efficiency for positively charged antimicrobial agents. Improved encapsulation can prolong the release profile of encapsulated antibiotics, with studies showing up to eightfold greater drug loading capacity than that of neutral liposomes (Hui et al., 2009; Messiaen et al., 2013).

The hydrophobic tail differs in acyl chain length, symmetry, and saturation (Large et al., 2021). Liposomal stability can be improved via lipids with longer tails and high degrees of tail saturation, i.e., lipids with high transition temperatures (T_m_); lipids with long saturated acyl chains exhibit stronger hydrophobic interactions, resulting in higher T_m_ than lipids with shorter or unsaturated chains do (Kohli et al., 2014). As a result, phospholipids with longer saturated hydrocarbon chains tend to form rigidly ordered bilayer structures (Guimarães et al., 2021; Kapoor et al., 2017). However, the length of the fatty acyl chain also significantly affects drug loading and encapsulation in liposomes, with shorter-chain lipids achieving higher encapsulation efficiency for hydrophilic drugs than longer-chain lipids do (Chang and Flanagan, 1995). Lipid chain length and saturation directly influence membrane fluidity, which can be a determinant of interactions with bacterial membranes. Liposomes composed of short-chained phosphatidylcholine and phosphatidylglycerol resemble bacterial phospholipid bilayers, facilitating membrane fusion and thereby enhancing the targeted delivery of encapsulated antimicrobial agents (Singh et al., 2024). Selecting suitable lipids and their ratios is essential, as these elements significantly influence properties such as particle size, rigidity, and surface charge, thereby determining the safety, stability, and efficacy of the liposomal product (Kapoor et al., 2017; Nsairat et al., 2022).

In addition to phospholipids, other components, such as cholesterol and polyethene glycol (PEG), can enhance liposomal stability and elimination half-life (Inglut et al., 2020). Adding cholesterol to liposomal membranes affects their fluidity and rigidity, thereby decreasing leakiness through the lipid bilayer and increasing their stability both in vitro and in vivo. Cholesterol can achieve this by inducing dense packing of phospholipids by intercalating between their fatty acyl chains, thus stabilising the liposomal bilayer (Bozzuto and Molinari, 2015b; Briuglia et al., 2015; Lee et al., 2005; Sharifi et al., 2019). Once in the bloodstream, however, the stability of liposomes is challenged, as plasma proteins adhere to the liposomal surface, leading to their rapid clearance via a process known as opsonisation. This phenomenon not only hinders drug delivery to the target site but also inflicts damage to organs where the drug accumulates (Li et al., 2019). The incorporation of PEGylated phospholipids into liposomes provides steric hindrance, thereby significantly inhibiting protein adsorption and prolonging liposome circulation time in the bloodstream (Large et al., 2021). PEGylation can be covalently attached to lipids, such as distearoyl phosphatidylcholine polyethene glycol 2000 (DSPE-PEG2000), a commonly used PEGylated phospholipid in liposomal formulations (Li et al., 2015). The incorporation of PEGylated phospholipids into liposomal surfaces can also significantly extend their blood circulation half-life, from a few minutes, as observed with conventional liposomes, to several hours, thus creating PEGylated (stealth) liposomes (Beltrán-Gracia et al., 2019). Extended circulation for PEGylated liposomes can increase their stability (Bulbake et al., 2017) and enable passive targeting at infection sites (Laverman et al., 2001), making them promising carriers for combating AMR. In addition, PEGylation can further enhance antimicrobial efficacy by reducing interactions between liposomes and efflux pumps, which are frequently observed in MDR bacterial strains (Ladva et al., 2024). PEGylation also improves the overall pharmacokinetics of liposomes while exhibiting minimal interaction with healthy cells in the body (Belfiore et al., 2018; Huwyler et al., 2008). This approach led to the success of Doxil® as an intravenous treatment for advanced ovarian cancer, multiple myeloma, and HIV-associated Kaposi's sarcoma (Bulbake et al., 2017). Another PEGylated liposomal formulation available on the market is Onivyde®, an irinotecan liposomal formulation. It has received FDA approval for treating metastatic pancreatic adenocarcinoma (FDA, 2015; Rahman et al., 2017). It consists of unilamellar liposomes approximately 110 nm in diameter (Mast et al., 2021). The vesicles are composed of DSPC, cholesterol, and DSPE-PEG2000 (Drummond et al., 2006; Kirpotin et al., 2023). Despite their advantages, PEGylated liposomes are not without challenges, as several limitations associated with PEGylation have emerged over time. Once thought to be biologically inert, PEGylated liposomes can trigger specific side effects by activating the complement system (Moghimi and Szebeni, 2003).

Furthermore, in comparison to the conventional liposomal antimicrobials, stimuli-responsive liposomal formulations have emerged as alternatives, enabling precise and controlled drug delivery activated by external triggers (heat, ultrasound, light) or internal stimuli (enzymatic activity, pH changes) at the disease site (Bibi et al., 2012). Among the internal stimuli, pH- and enzyme-responsive liposomes have shown notable potential and clinical relevance for antimicrobial therapy (Devnarain et al., 2021). For example, bacterial infections typically generate acidic microenvironments due to anaerobic metabolism and inflammatory responses. A pH-triggered responsive system can enhance uptake by negatively charged bacterial cell walls in both gram-positive and gram-negative bacteria (Radovic-Moreno et al., 2012). Such intrinsic stimuli-responsive approaches can enhance site-specific drug delivery, reduce systemic toxicity, and potentially mitigate AMR by ensuring higher drug concentrations at infection sites (Canaparo et al., 2019; Devnarain et al., 2021). Another commonly explored type of stimuli-responsive liposome is temperature-sensitive liposomes (TSLs) (Li et al., 2010), which retain their payload at body temperature but release it upon exposure to local heat (Nisini et al., 2018a). However, TSLs have been predominantly developed and utilised for cancer therapy rather than for targeting AMR.

## 6 Commercial manufacturing methods for liposomal antimicrobials

**Fig. 5:**
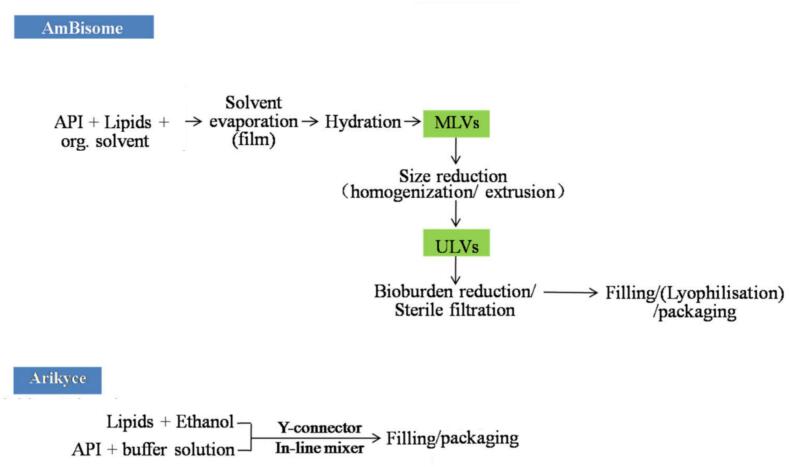
Commercial manufacturing processes for AmBisome® and Arikayce® liposomal drug products. *Abbreviations: API: active pharmaceutical ingredients, MLVs: multilamellar vesicles, ULVs: unilamellar vesicles.

Translating the liposomal antimicrobials into clinically approved products strongly depends on developing robust, scalable, and economically viable manufacturing techniques. Meeting these demands requires flexible production platforms integrated with rigorous quality control measures at every stage of the manufacturing process (Biscaia-Caleiras et al., 2024). While several methods exist for manufacturing liposomes at the laboratory scale, only a few are employed in commercial production to ensure that liposomes meet the necessary critical quality attributes (Biscaia-Caleiras et al., 2024; Shah et al., 2020). Of the various liposomal preparation methods described in the literature, only a limited number are amenable to industrial-scale manufacture. In particular, the ethanol injection / in-line infusion method used for Arikayce® represents a scalable, continuous process suitable for large-volume production (Fig. 5), whereas laboratory-based techniques such as thin-film hydration/solvent evaporation, reverse-phase evaporation and microfluidics are generally not scalable and are mainly restricted to research settings (Kapoor et al., 2017; Liu et al., 2022; Lombardo and Kiselev, 2022). AmBisome® is produced using a proprietary, modified solvent-evaporation and spray-drying process (Fig. 5) that is distinct from conventional thin-film hydration and is specifically adapted for industrial GMP manufacturing (Rivnay et al., 2019). The standard manufacturing steps typically consist of (i) forming either multilamellar or unilamellar vesicles, depending on the method used; (ii) size reduction, if needed; (iii) preparing and loading the drug solution, occasionally merging this with the initial vesicle formation for passive loading; (iv) performing buffer exchange and concentration, as needed; (v) conducting sterile filtration or aseptic processing; and (vi) lyophilisation followed by packaging (Liu et al., 2022). The following sections provide detailed manufacturing protocols for AmBisome® and Arikayce®.

### 6.1 Patented method of manufacturing AmBisome®

**Fig. 6:**
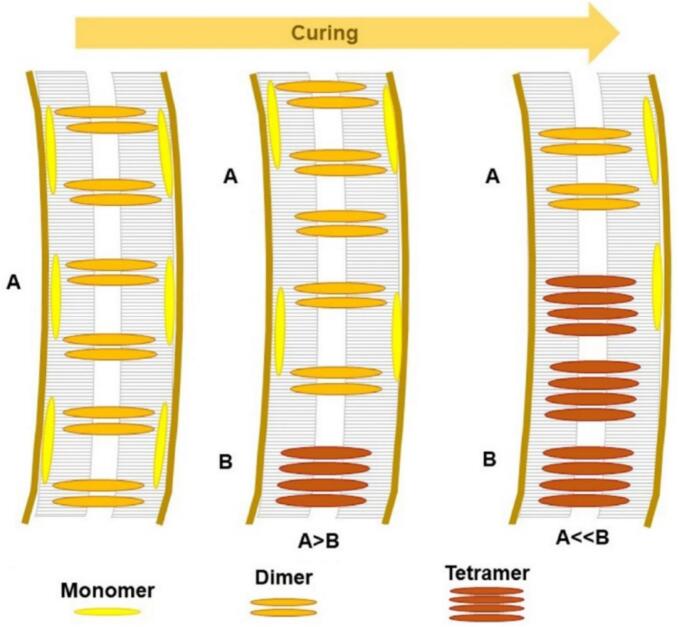
Illustration of AmB organisation within the lipid bilayer during curing. Curing induces a shift from loosely associated monomers/dimers (left) to more ordered and stable higher-order aggregates (right), such as tetramers, enhancing bilayer stability and lowering toxicity; adapted from Svirkin et al. (2022).

In the film hydration solvent evaporation method, lipids and hydrophobic drugs are dissolved in an organic solvent (often a mixture of chloroform and methanol; others can be ether, ethanol, or dichloromethane). The solvent is subsequently removed under reduced pressure to form a thin film. The thin film is then hydrated with aqueous buffer by stirring it at a temperature above the phase transition of the lipid, resulting in the formation of liposomes with multilamellar structures, which require further processing, i.e., downsizing via homogenisation to control vesicle size and lamellarity (Hu et al., 2021; Zhang, 2017). The original manufacturing protocol for AmBisome® was obtained from Proffitt et al., 1999 (Proffitt et al., 1999). DSPG is mixed with a chloroform-methanol solution that has been prewarmed to 65 °C. The mixture is then acidified with HCl, and AmB is suspended in the same solvent to form a translucent solution. Separate solutions of HSPC and cholesterol in a similar solvent mixture, also prewarmed to 65 °C, are combined with the drug solution. After sufficient mixing to achieve a homogeneous solution, sodium hydroxide was used to balance the pH. The organic solvent was then removed by spray drying, resulting in a dry, yellow–brown powder. This powder was hydrated with a prewarmed lactose solution in sodium succinate buffer. The hydrated dispersion is subjected to a shearing force, which reduces the particle size, resulting in unilamellar vesicles. Finally, the liposomal dispersion is lyophilised and can be reconstituted in water before administration. This multistep manufacturing process for AmBisome® is crucial and can impact the fundamental physicochemical and toxicological properties of the final drug product (Rivnay et al., 2019). Acidification by HCl is a crucial step in facilitating the dissolution of DSPG in the organic solvent mixture and promoting the initial formation of drug–lipid complexes (Rivnay et al., 2019; Watts et al., 1978). At the molecular level, acidification likely neutralises the negatively charged phosphate group of DSPG, converting it from an inverse micellar arrangement to a freely soluble lipid monomer form. The neutral form of DSPG can then effectively associate with AmB, overcoming the charge-based barriers that would otherwise hinder complex formation at this stage (Rivnay et al., 2019). Excessive addition of HCl should be avoided, as it can cause AmB degradation in the solvent mixture (Rivnay et al., 2019). Following homogenisation (the downsizing step), the liposomal dispersion is heated (cured) above the lipid transition temperature, typically approximately 65 °C, to optimise the drug-lipid interactions. This curing step is thought to influence the molecular arrangement of AmB within the lipid bilayer, forming tightly packed super-aggregates that release the drug more slowly and reduce toxicity during systemic circulation (Rivnay et al., 2019; Svirkin et al., 2022). The reduced toxicity is closely linked to the slower dissociation of these tight aggregates, i.e., AmB tetramers, which limits the immediate availability of monomeric or dimeric AmB — the species primarily associated with membrane disruption and toxicity (Fig. 6) (Svirkin et al., 2022; Wasko et al., 2012).

AmBisome® undergoes sterilisation during manufacturing through filtration of the bulk liposomal dispersion, followed by aseptic lyophilisation (Naik et al., 2017; Proffitt et al., 1999), which ensures sterility without compromising liposomal structure or drug–lipid complex integrity. However, Manufacturing stresses such as sterile filtration and lyophilisation can lead to issues with liposomes, including drug leakage, phase separation, and disintegration. As a result, monitoring for any such changes throughout each stage of manufacturing is essential to ensure batch reproducibility (Adler-Moore et al., 2016). Additionally, even slight changes in the molar ratios of AmB to lipids such as HSPC and DSPG, as well as modifications in the fatty acid chain length of phosphatidylglycerol, can significantly influence the association between AmB and the liposome bilayer. These variations can substantially impact the toxicity of LAmB formulations in mice, demonstrating the critical nature of precise manufacturing control (Adler-Moore et al., 2016; Adler-Moore and Proffitt, 1993).

Several generic LAmBs have been previously approved outside the United States, although some, including Anfogen® (Genpharma, Argentina) and Lambin® (Sun Pharmaceuticals Ind. Ltd., India), have been withdrawn from the market because their toxicity (Kshirsagar, 2014) exceeds that of AmBisome®, despite having the same chemical composition (Olson et al., 2008; Olson et al., 2014). The significant differences in the toxicity profiles of AmBisome® and these generics highlight the critical importance of ensuring quality and uniformity across different products and within batches. Manufacturing methods that produce polydisperse liposomes may lead to faster drug release and increased toxicity in clinical applications (Liu et al., 2020b). Despite the expiration of the AmBisome® US patent in 2016 (Liu et al., 2020b), only two generic versions of LAmB have been approved by the FDA (Pharma, 2022; Sun Pharma, 2021) and one by the UK Medicines and Healthcare Products Regulatory Agency (MHRA, 2024). Given the significant differences in toxicity observed with some earlier, withdrawn generics of AmBisome®, clear regulatory guidance is critical to ensure the quality and safety of LAmB products. In contrast, the more recent FDA- and MHRA-approved generics have fulfilled current guidance requirements, which rationalise the need for stringent evaluation criteria. In this context, the FDA, EMA, and WHO have published comprehensive guidelines on bioequivalence, evaluation, and dosing to ensure quality and uniformity across different products and batches of LAmB (EMA, 2013; FDA, 2020a; Lee et al., 2024). Nevertheless, the intricate structure and process dependence of LAmB indicate that rigorous control of manufacturing is capital-intensive at a commercial scale.

### 6.2 Original manufacturing method of Arikayce®

Generally, the solvent injection method involves dissolving lipids and hydrophobic materials in a water-miscible organic solvent, such as ethanol, and then injecting this solution into an aqueous phase with continuous stirring. This process results in the spontaneous formation of large or small unilamellar vesicles (Large et al., 2021; Sala et al., 2017). Arikayce® utilises a modified ‘ethanol infusion’ or ‘in-line infusion’ technique to prepare AMK liposomes, which differs slightly from the conventional ethanol infusion process (Fig. 5) (Boni et al., 2014; Perkins et al., 2017). DPPC and cholesterol were dissolved at a 2:1 weight ratio in absolute ethanol. AMK sulfate is solubilised in sterile, deionised water, and the pH is adjusted with NaOH. The lipids in ethanol and AMK in aqueous solution are then infused through a Y-connector and an inline mixer at a controlled moderate temperature (∼30–40 °C) and with differential flow rates (∼ 300 mL/min for lipids and 500 mL/min for AMK), which promotes rapid mixing and efficient drug capture. The mixed stream is subsequently combined with sodium citrate buffer, and the resulting liposomal dispersion is quickly diluted with sodium chloride (NaCl); unencapsulated drug and residual solvent are then removed via tangential flow filtration (Boni et al., 2014; Li et al., 2008; Perkins et al., 2017; Zhang et al., 2018). Owing to its limited solubility in ethanol, AMK adopts a transient, semi-coacervate state under these ethanol-rich, moderate-temperature, fast-mixing conditions, facilitating its entrapment within the liposomal aqueous core (Zhang et al., 2018), which in turn enables very high encapsulation (≤ 5 % unencapsulated drug; i.e., ∼ 95–98 % encapsulation efficiency) and a lipid-to-drug mass ratio of ∼0.7 (Boni et al., 2014). The in-line infusion method used in Arikayce® manufacturing produces liposomes with mean diameters of 250–300 nm and requires no further processing, e.g., extrusion or homogenisation, to control the size distribution (Li et al., 2008). The high ethanol content in the lipid mixture necessitates careful management to prevent AMK precipitation, which can be achieved by adding NaCl either during or after the infusion process. Liposomes are assumed to have already formed when NaCl is added (Boni et al., 2014). The retention of AMK within the liposomal aqueous core involves further control of osmolality by adjusting the NaCl concentration across the liposome membranes (EMA, 2020b). Due to its multicationic nature, once AMK is encapsulated within liposomes, it shows low permeability through the liposome membrane, resulting in stable suspensions even at room temperature. (Liu et al., 2022). Arikayce® is supplied as a sterile liposomal suspension produced entirely by aseptic processing, rather than through terminal sterilisation (EMA, 2016). Regulatory documentation specifies that the product is manufactured using sterile raw materials, sterile equipment and controlled Class A environments (EMA, 2019), because its ∼300 nm liposomes cannot withstand heat, irradiation or membrane filtration without compromising structural integrity or drug loading. Aseptic processing is therefore necessary for sterile medicines that cannot be sterilised using conventional methods, ensuring the formulation stays sterile throughout preparation and filling (Delma et al., 2021; Galante et al., 2016). Overall, the manufacturing process for Arikayce® was designed to ensure a robust method of production, taking into account the physicochemical and biological characteristics of the product (EMA, 2020b).

### 6.3 Challenges in current liposomal manufacturing methods

Liposomes have been extensively investigated as specialised drug delivery systems that offer benefits such as targeted delivery, protection of encapsulated agents from degradation, enhanced solubility and bioavailability, and improved safety and efficacy (Luo et al., 2025; Shah et al., 2020). Despite the increasing number of liposomal products available, the development and translation of clinically successful liposomal drugs involves a range of challenges, including manufacturing and scalability issues, maintaining stability during production and storage, achieving effective sterilisation without compromising liposome integrity, and complying with strict regulatory requirements. In addition, significant sustainability issues persist in the current commercial manufacturing of liposomal nanomedicines, limiting the wider accessibility and affordability of these advanced therapeutics and preventing them from reaching their full potential globally (Zhang et al., 2025).

### 6.4 Complexity and centralised manufacturing challenges

Traditional manufacturing approaches pose significant challenges to scaling up liposomal nanomedicine production, hindering clinical development due to substantial capital investment and ongoing running costs at commercial scales (Shah et al., 2020). Many liposomal preparation techniques are not easy to scale from the laboratory to commercial production because of their complexity and the lack of predictability and reproducibility of the resulting formulations (Jensen and Hodgson, 2020; Maja et al., 2020). Reducing the particle size to the submicron range, specifically 50–200 nm, is necessary, as it influences the pharmacokinetic and pharmacodynamic profiles of the particles in the body, thereby affecting the safety and efficacy of the final product (Gabizon et al., 2003; Janknegt et al., 1992). All techniques used to reduce liposome particle size have advantages and disadvantages. Sonication is a quick method for reducing particle size; however, it generates heat, which can cause peroxidative damage and lipid degradation (Shah et al., 2020). Additionally, probe sonication risks overheating, potentially contaminating the sample with titanium (Maja et al., 2020). Extrusion allows reproducible downsizing of the final liposome product (Ong et al., 2016); however, it is a labour-intensive and time-consuming process (Shah et al., 2020) with scalability challenges and issues such as membrane filter clogging and filter fragility. Homogenisation is a commonly used technique for downsizing and controlling particle size distribution, making it suitable for large-scale manufacturing. The significant drawbacks of this method include a broad and variable liposome size distribution, as well as potential contamination from metals and oil due to the use of very high pressure (Lombardo and Kiselev, 2022).

When combined, these complex batch processes in a commercial manufacturing setup, a centralised model dominated by large-scale facilities is inevitable. This model often requires upfront investments of hundreds of millions of dollars, high ongoing maintenance costs and complex supply chains. These facilities are typically located in a few high-capital-intensive sites, often in high-income countries, to produce a limited volume of high-value medicines. This centralised model creates significant barriers for generic manufacturers, reinforcing market concentration and exposing supply chains to vulnerabilities during disruptions such as pandemics or geopolitical tensions. For example, LAmB's global availability remains restricted, even after more than 30 years on the market, particularly in LMICs. The global annual supply shortage exceeds 9 million vials, which directly leads to high cost per treatment for patients (up to £50,000 per patient course in some regions) (MSF, 2022). Setting up one LAmB commercial manufacturing facility requires a high initial capital investment (over $200 million), and even when these facilities are fully operational, they produce limited quantities of LAmB (5 million vials/year) (Palmer, 2017). The scarcity of affordable generic (non-patented) supply exacerbates this problem, as this limited supply prevents competitive pricing. For example, a newly approved generic sells at the same price as the original in the UK (£80 per vial) (BNF, 2025). Theoretically, generics could alleviate these issues, but even after two US FDA approvals, the prices remain prohibitively high, up to $200/vial in private markets ($50,000 per patient course), and £80/vial in the UK (matching the original price in the NHS). Replicating traditional multi-step production processes only compounds costs, perpetuating monopolistic structures that prioritise profit over equity in underserved regions.

In contrast, a decentralised model can empower local and regional stakeholders and foster stronger resilience and accessibility for LMICs. This model enables the distribution of smaller, modular hubs across regions, facilitated by continuous and portable manufacturing platforms. These hubs can allow production to be scaled on demand. By partially or fully localising supply chains, these hubs can reduce dependencies on global logistics while promoting affordability, resilience and responsiveness to local health needs. This paradigm shift aligns with international initiatives, such as WHO/Unitaid's World Local Production Forum, and a recent $50 million investment in African regional manufacturing, which emphasises their commitment to affordable access.

An additional manufacturing challenge arises from the use of organic solvents in liposome preparation, particularly for intravenous formulations (Panthi et al., 2024). Common examples include chloroform and dichloromethane, both of which are classified as Class II solvents by the ICH Q3C guidelines due to their inherent toxicity (ICH, 2024). Residual traces of these solvents may not be entirely eliminated from the final product, posing a risk of toxicity through various mechanisms (Alhajlan et al., 2013; Maja et al., 2020; Marchianò et al., 2020). Removing these residual solvents typically requires additional processing steps, that can complicate scalability and increase manufacturing complexity and costs (Lombardo and Kiselev, 2022).

### 6.5 Stability and sterilisation

Stability issues in liposomal formulations originate from multiple sources, including formulation composition, manufacturing processes, and storage conditions. Chemical and physical degradation processes can critically limit liposomal stability, as they can shorten the shelf-life of products (Storm and Crommelin, 1998). Lipid degradation during manufacturing and storage represents a critical stability issue for liposomal products. Lipids are inherently susceptible to hydrolysis and oxidation, processes that generate degradation products such as lysolipids. These lysolipids significantly impact the safety and efficacy of liposomes, potentially causing cytotoxic effects, including haemolysis, apoptosis, and increased membrane permeability, even at low concentrations (Kapoor et al., 2017; Li et al., 2015; Toh and Chiu, 2013). Furthermore, lysolipid accumulation reduces colloidal stability and negatively affects the encapsulation efficiency of therapeutic cargo molecules, thereby impairing the biological performance of the final product (Lombardo and Kiselev, 2022). Given these risks, thorough characterisation and strict control of lipid degradation products, in compliance with ICH guidelines (ICH, 2006), are necessary for maintaining liposomal formulation stability and safety. Ensuring the stability of these lipid vesicles in aqueous dispersions requires evaluating the chemical integrity of lipids and drugs, as well as the physical stability of the vesicles, including their size and shape (Monteiro et al., 2014; Sainaga Jyothi et al., 2022).

The chemical stability of liposomal formulations largely depends on their lipid composition, as lipids are susceptible to chemical reactions such as hydrolysis of ester bonds and oxidation of unsaturated fatty acyl chains. These reactions involve both synthetic and natural phospholipids (Sharma and Sharma, 1997; Storm and Crommelin, 1998). Furthermore, assessing the chemical stability of the encapsulated drug could also be considered (Lin et al., 2021). These chemical reactions can impact the structural integrity of liposomes, resulting in alterations in lipid membrane permeability (Large et al., 2021). The physical stability of liposomal formulations is influenced by vesicle aggregation, fusion, and leakage of the encapsulated drug (Monteiro et al., 2014). Fusion occurs when smaller vesicles merge to form larger ones, typically in an irreversible manner, whereas aggregation involves the agglomeration of vesicles without fusion.

The stability of liposomal drugs depends on multiple factors, including lipid composition, manufacturing method, pH, and storage conditions (Drulis-Kawa and Dorotkiewicz-Jach, 2010; Storm and Crommelin, 1998). Chemical instability, particularly oxidation and hydrolysis, can be mitigated by incorporating antioxidants such as butyl hydroxytoluene and α-tocopherol to protect phospholipids from oxidative degradation (Sainaga Jyothi et al., 2022). Furthermore, excluding oxygen from injection vials, selecting saturated acyl-chain phospholipids, and maintaining an environmental pH of 6.5 at low storage temperatures further prevent oxidation and hydrolysis (Storm and Crommelin, 1998; Zuidam et al., 1995). Physical stability concerns, such as vesicle aggregation, fusion, and leakage, can be addressed by modifying the liposomal surface. Coating liposomes with polymers such as PEG enhances their stability through steric hindrance, reducing unwanted vesicle interactions (Sainaga Jyothi et al., 2022). If these conditions cannot be ensured, freeze-drying in the presence of stabilising cytoprotectants can help preserve lipid integrity and minimise hydrolytic degradation (Ahmed et al., 2019). Furthermore, storing liposomal formulations in a dry state, rather than in aqueous dispersions, prevents physical destabilisation and prolongs shelf-life (Chen et al., 2010). Freeze-drying can effectively preserve liposomes if suitable cryoprotectants and optimised conditions are used. Disaccharides, as effective cryoprotectants, prevent vesicle aggregation and fusion upon rehydration. While some drug leakage may still occur after the freeze–drying–rehydration cycle, advances in cryoprotection strategies have improved the stability and retention of encapsulated drugs (van Winden and Crommelin, 1997).

For a pharmaceutical product, a minimum shelf life of two years, preferably without refrigeration, is a primary requirement (Storm and Crommelin, 1998). Beyond physical and chemical stability, the preferred storage conditions for commercial liposomal products play a fundamental role in determining the final dosage form. While room-temperature storage is ideal for logistical and economic reasons, most marketed liposomal formulations require refrigeration (2–8 °C) due to stability challenges. This is applicable for Arikayce®, which must be stored at 2–8 °C for up to 3 years, although it can be kept at room temperature for up to 4 weeks (FDA, 2018a). AmBisome®, on the other hand, is supplied as a lyophilised product with a relatively long shelf life (4 years) and can be stored at room temperature (< 25 °C) for the full duration (Gilead, 2024). Particle size, drug loading, and chemical integrity can be significantly affected when liposomes are stored as liquid dispersions at room temperature. Freeze-drying or spray-drying liposomal dispersions into solid formulations can enhance stability by minimising interactions between drug components. However, this approach has limitations, including changes in particle size after reconstitution and reduced entrapment efficiency for hydrophilic drugs or actively loaded compounds, such as doxorubicin. As a result, liquid formulations stored at 2–8 °C remain the preferred option in many cases, even for dried formulations. Additionally, converting liposomes into solid dosage forms requires extra manufacturing steps and in-process quality control, and choosing between liquid and dry formulations is dependent on product requirements, target markets, and storage capabilities (Shah et al., 2020).

Moreover, sterilisation during the filling/finishing process of liposomal nanomedicine may also affect its stability. Sterilisation is a crucial aspect of injectable formulations, as most liposomal drug products are designed for parenteral administration. However, this process remains challenging because of the sensitivity of lipids and their physicochemical instabilities (Sainaga Jyothi et al., 2022). Conventional sterilisation methods, such as heat treatment (steam and dry heat), irradiation (including gamma and ultraviolet), or chemical agents (ethylene oxide), are generally unsuitable because of the sensitivity of lipids to high temperatures, oxidation, and hydrolysis (Araki et al., 2018; Zuidam et al., 1993). Conventional sterilisation methods, such as heat treatment (steam and dry heat), irradiation (including gamma and ultraviolet), or chemical agents (ethylene oxide), are generally unsuitable due to the sensitivity of lipids to high temperatures, oxidation, and hydrolysis (Araki et al., 2018; Zuidam et al., 1993). Autoclaving is a feasible option for sterilising liposomes, but it is only applicable when the drug is heat-stable, lipophilic, and the pH conditions are optimal. In cases where heat sterilisation is not viable, alternative aseptic production methods, including filtration through 0.2 μm membranes, are needed (Storm and Crommelin, 1998). Mechanical filtration is commonly used, particularly for liposomal vesicles smaller than bacterial cells. However, this method does not entirely eliminate viral contaminants, limiting the ability to achieve complete sterility (Sharma and Sharma, 1997). For most liposomal antibiotics, filtration or aseptic processing is necessary to ensure product safety (Storm and Crommelin, 1998; Zuidam et al., 1993). Endotoxin removal is also crucial for all parenteral nanoparticle formulations before their administration to humans. The Limulus amoebocyte lysate (LAL) test is the most widely used method for detecting endotoxins in drug products and must be thoroughly validated for pharmaceutical liposomes (Sainaga Jyothi et al., 2022; Storm and Crommelin, 1998; Vetten et al., 2014). However, recombinant Factor C (rFC) assays—an animal-material-free alternative—are now recognised in compendial standards (e.g., USP Chapter <86>) and recent studies demonstrate their equivalence to LAL methods (Bolden and Smith, 2017; Kang et al., 2024; USP, 2024). While standard pyrogen-free production procedures can be applied, endotoxins pose a challenge as they can pass through 0.22 μm membrane filters. To address this, pharmaceutical industries employ additional filtration techniques, such as chromatography, gel electrophoresis, gel filtration, and ultrafiltration, to ensure endotoxin-free formulations (Magalhães et al., 2007).

### 6.6 Quality assurance and regulatory considerations

As outlined in FDA guidance, liposomal products are complex medicines that are susceptible to variations in manufacturing conditions, including changes in processing approaches and batch scale. It is essential to establish robust process controls during the product development process. Applying **quality-by-design (QbD)** principles in the early stages of product development enables the identification and implementation of appropriate controls. Integrating QbD throughout development helps establish a **design space**, allowing flexibility in selecting formulation and manufacturing parameters to achieve the desired quality attributes of a drug product. Additionally, QbD can facilitate the scalability of liposomal formulations, addressing one of the significant challenges in their development (Chen et al., 2010; Kapoor et al., 2017). Utilising existing knowledge and conducting risk assessments can help identify critical process parameters (CPPs) that impact the quality of the final product (FDA, 2018b). Therefore, irrespective of the chosen manufacturing process for liposomes, understanding the impact of CPPs on product quality and safety is fundamental. Through prior knowledge and/or risk assessment techniques, manufacturers can ensure a robust process that delivers consistent batch-to-batch reproducibility and maintains product quality, even with minor deviations in process parameters (Kapoor et al., 2017). Since the release of EMA reflection papers (EMA, 2011), regulatory guidance for complex generics such as LAmB has advanced considerably, with more recent progress exemplified by the FDA's product-specific guidance (FDA, 2020) and the 2025 WHO notes on LAmB bioequivalence (BE) study design (WHO, 2025). The FDA guidance requires sameness in Q1/Q2 and comprehensive in vitro and in vivo comparability assessments (FDA, 2020), whereas the EMA reflection paper emphasises detailed characterisation and, in some cases, nonclinical or clinical studies to confirm therapeutic equivalence (e.g., LAmB distributions in the objects of interest). Although manufacturing complexity remains a significant hurdle, these developments illustrate the evolution of the regulatory landscape, which has now enabled the approval of LAmB generics by both the FDA and MHRA. Table 3 summarises the current guidance from the FDA, EMA, and WHO, including key requirements for composition, characterisation, and bioequivalence assessment.

**Table 3:** Side-by-Side comparison of the regulatory requirements for liposomal amphotericin B (LAmB) from different agencies.

Agency	Key Document (Year)	Focus/Requirements
FDA	Product-Specific Guidance: LAmB injection (Revised 2020)	• Requires Q1/Q2 sameness (qualitative/quantitative excipient equivalence) • Comparative in vitro liposome characterisation (size distribution, lamellarity, bilayer phase transition, charge, leakage rates) • In vivo BE study: single-dose, crossover design with PK endpoints; encapsulated AmB as primary parameter, free drug supportive
EMA	Reflection Paper on IV liposomal products (2011)	• Emphasises detailed characterisation of liposomes (size, morphology, surface charge, composition, stability) • Pharmaceutical comparability is essential but cannot replace nonclinical/clinical data • Case-by-case: may require additional PK, PD, or clinical studies to confirm therapeutic equivalence, perhaps in disease models
WHO	PQT Guidance Notes: Bioequivalence study design for LAmB (2025)⁎	• Requires Q1/Q2 sameness and liposome comparability (morphology, size distribution, lamellarity, charge, leakage rates) • Single-dose crossover PK study • BE endpoints: encapsulated AmB (primary), unencapsulated AmB (supportive, incl. Partial AUCs) • If excipient composition differs, a full comparability exercise is necessary (quality, nonclinical, clinical)

⁎ WHO guidance refers specifically to the Prequalification Programme (PQT) notes on liposomal amphotericin B (2025). No general WHO framework currently exists for nonbiological complex drugs (NBCDs), such as liposomes.

## 7 Future perspectives on liposomal antimicrobials

Bacterial and fungal infections constitute a growing global health crisis, exacerbated by the rapid emergence and spread of AMR. Liposomal nanoformulations can transform antimicrobial treatment by addressing crucial therapeutic limitations, including high drug toxicity, poor solubility, and barriers posed by resistant pathogens. The clinical success of AmBisome® and Arikayce® demonstrates the importance of liposomes and highlights their enhanced efficacy, targeted delivery, improved safety profiles, and ability to penetrate infection sites, including biofilms and mucosal interfaces. The lessons learned from anticancer liposomal nanomedicines highlight the versatility of nanocarriers: customised compositions, surface modifications, and stimuli-responsive systems can help us improve antimicrobial therapies. Liposomal designs for antibiotic delivery can draw directly from anticancer strategies, as the infection sites and tumours share key microenvironmental features that influence drug distribution, tissue penetration, and therapeutic efficacy (Goldszmid et al., 2014; Yusuf et al., 2023). Both settings display abnormal, highly permeable vasculature arising from angiogenic dysregulation in tumours and inflammation-induced endothelial disruption in microbial infections (Islam et al., 2022). They also contain hypoxic, poorly perfused regions, whether due to vascular disorder in tumours or high microbial metabolic demand in biofilms, which limit drug penetration and alter pharmacodynamics (Sønderholm et al., 2017; Wang et al., 2022). In addition, both environments possess dense extracellular matrices, from fibrotic stroma in tumours to polysaccharide-rich biofilms in infections, that restrict nanoparticle diffusion (Eble and Niland, 2019; Flemming and Wingender, 2010). Finally, these microorganisms' ability to alter the host immune system, including tumour-mediated immunosuppression, pathogen-driven biofilm shielding, and intracellular persistence, can create further barriers to effective therapy (Arciola et al., 2018). Thus, we believe that oncology-derived liposomal nanocarrier design principles may offer established features to support the development of new liposomal antimicrobials.

Furthermore, beyond the clinical success of currently marketed liposomal products such as AmBisome® and Arikayce®, other antibiotic classes could be prioritised for liposomal reformulation, particularly when high toxicity and suboptimal tissue penetration limit current treatments. Polymyxins, particularly colistin (polymyxin E) and polymyxin B, are classified by WHO as Highest Priority Critically Important Antimicrobials (WHO, 2023). Its significance has grown over the past decade, as it remains one of the few available options for treating multidrug-resistant organisms (Anderson et al., 2022). It now serves as salvage therapy for MDR *P. aeruginosa*, *Klebsiella* spp., and *A. baumannii* in ventilator-associated pneumonia, sepsis, cystic fibrosis, and burn wound infections, where therapeutic options are limited. (Abesamis and Cruz, 2019; Jalil et al., 2024; Vairo et al., 2023). However, their clinical use is severely limited by dose-dependent nephrotoxicity and neurotoxicity, particularly with intravenous colistin (Huang et al., 2024; Li and Schneider-Futschik, 2023; Vairo et al., 2023). As polycationic, amphiphilic peptides, polymyxins interact strongly with anionic bacterial membranes, they can also be effectively incorporated into anionic lipids, where opposite charge pairing supports efficient liposomal encapsulation and controlled release (Dubashynskaya and Skorik, 2020). In vitro, liposomes containing polymyxin B have demonstrated 4- to 16-fold reductions in MICs against multiple Gram-negative pathogens (including *P. aeruginosa*, *K. pneumoniae*, *and A. baumannii*) (Drulis-Kawa and Dorotkiewicz-Jach, 2010). Intravenous polymyxin B-loaded liposomes in rodent models increase lung drug levels, reduce inflammatory markers, and markedly improve control of *P. aeruginosa* lung infection, while minimal kidney and serum exposure indicates a substantially lower risk of systemic toxicity (Alipour and Suntres, 2014). Also, colistin is a strong candidate for liposomal pulmonary delivery because inhalation can slow resistance development, and liposomal formulations enhance lung deposition while reducing nephrotoxicity (Yu et al., 2020). Together, these data highlight polymyxins as a prominent antibacterial class with the potential to match the clinical success of liposomal AMK, provided that formulation stability and large-scale manufacturing challenges are addressed. Other antibiotic classes, such as glycopeptides, may also benefit from liposomal reformulation. In particular, vancomycin is a plausible liposomal candidate, as this first-line agent for MRSA is limited by nephrotoxicity and by poor penetration into bone tissue and mature biofilms due to its high molecular weight and hydrophilicity (Scriboni et al., 2019). Liposomal vancomycin has shown prolonged circulation, enhanced killing of MRSA within macrophages, reduced renal accumulation, and improved activity against *S. aureus* biofilms and osteomyelitis models compared with the free drug (Ferreira et al., 2021a; Scriboni et al., 2019). Collectively, these findings support the notion that liposomal delivery of glycopeptides, such as vancomycin, can optimise pharmacokinetic profiles, reduce toxicity, and enhance efficacy at hard-to-reach sites of gram-positive infections.

However, substantial hurdles remain to bringing liposomal antimicrobials to broader clinical use. Challenges related to manufacturing processes, scalability, physical and chemical stability, and regulatory requirements may hinder widespread commercial adoption and translations in LIMCs. Additionally, the balance between advanced functionalities and cost-effective, reproducible manufacturing technologies remains a significant barrier. Future research should prioritise simplifying and streamlining the design and manufacturing of liposomes to improve their reproducibility, scalability, and affordability. Such efforts would not only accelerate the clinical translation of innovative antimicrobial liposomal products but also ensure their global accessibility and sustainable use in resource-constrained regions. Advancing the technological, regulatory, and economic aspects of liposomal antimicrobial development will be crucial in addressing the significant global health challenge posed by AMR.

## CRediT authorship contribution statement

**Hussein T. Kenaan:** Writing – review & editing, Writing – original draft, Investigation, Formal analysis, Conceptualization. **Ross M. Duncan:** Writing – review & editing, Writing – original draft, Formal analysis. **Wafa T. Al-Jamal:** Writing – review & editing. **David S. Jones:** Writing – review & editing. **Gavin P. Andrews:** Writing – review & editing, Project administration. **Brendan Gilmore:** Writing – review & editing. **Vanessa Yardley:** Writing – review & editing. **Nicola Farrington:** Writing – review & editing. **Katharine E. Stott:** Writing – review & editing. **David Lawrence:** Writing – review & editing, Conceptualization. **Joseph N. Jarvis:** Writing – review & editing, Conceptualization. **Thomas S. Harrison:** Writing – review & editing, Conceptualization. **Stephen Robinson:** Writing – review & editing, Conceptualization. **Isabela Ribeiro:** Writing – review & editing, Conceptualization. **William Hope:** Writing – review & editing, Project administration, Conceptualization. **Yiwei Tian:** Writing – review & editing, Writing – original draft, Supervision, Project administration, Funding acquisition, Formal analysis, Conceptualization.

## Declaration of competing interest

The authors declare that they have no known competing financial interests or personal relationships that could have appeared to influence the work reported in this paper.

## Data Availability

No data was used for the research described in the article.
